# Host‐Guest Synergistic Regulation in Functionalized Metal‐Organic Frameworks for Efficient Aqueous Zinc‐Ion Batteries

**DOI:** 10.1002/advs.202511198

**Published:** 2025-08-11

**Authors:** Yanfei Zhang, Qian Li, Yichun Su, Yangyang Sun, Shuai Cao, Shengjie Gao, Haotian Yue, Hsiao‐Chien Chen, Huan Pang

**Affiliations:** ^1^ School of Chemistry and Chemical Engineering Yangzhou University Yangzhou Jiangsu 225002 P. R. China; ^2^ College of Chemistry and Chemical Engineering Chongqing University of Science and Technology Chongqing 401331 P. R. China; ^3^ School of Environmental Science Nanjing Xiaozhuang University Nanjing Jiangsu 211171 P. R. China; ^4^ Center for Reliability Science and Technologies Kidney Research Center Department of Nephrology Chang Gung Memorial Hospital Chang Gung University Linkou Taoyuan 333 Taiwan

**Keywords:** aqueous zinc ion batteries, polyoxometalate, reaction mechanism, synergistic effect, vanadium‐based MOFs

## Abstract

Vanadium‐based metal‐organic framework (V‐MOF) cathodes in aqueous zinc‐ion batteries (AZIBs) are prone to be pronounced volumetric expansion during charging/discharging processes, which causes structural collapse, capacity fading, and compromised cycling stability. Herein, a series of novel hybrid nanomaterials (Br@P‐X) are successfully prepared via a one‐step solution method by confining polyoxometalates (POMs) within the pores of the V‐MOF and precisely controlling the POMs loadings. The synergistic structural and functional interactions between the porous MOF framework and the uniformly dispersed POMs, as well as the precise tuning of guest POM clusters endow the system with unique electrochemical behavior. The Br@P‐16 cathode possesses excellent structural and chemical stability, thus demonstrating outstanding electrochemical performance in AZIBs. The coordination environment of Br@P‐16 is investigated using X‐ray absorption fine structure spectroscopy. In‐situ X‐ray diffraction and ex‐situ X‐ray photoelectron spectroscopy/Fourier transform infrared analyses reveal the structural evolution during the electrochemical cycling process. This study provides a novel perspective for the design and synthesis of high‐performance cathode nanomaterials for AZIBs through the confinement strategy.

## Graphical abstract

This study proposes a sub‐nanometer cluster‐confinement strategy to confine polyoxometalate clusters within MOFs pores with precise loading control, enabling systematic elucidation of the synergistic mechanisms among sub‐nanometer clusters, non‐covalent interactions, and the porous framework in enhancing AZIBs performance. By integrating XAFS and in situ/ex situ spectroscopic techniques, the Zn^2+^ storage mechanism, charge‐distribution dynamics, and structural evolution throughout cycling are elucidated.

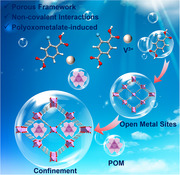

## 1 Introduction

The continuous growth of global energy demand and the increasingly serious environmental issues have driven the rapid development of low‐cost, high‐safety energy storage and conversion systems.^[^ ^1^ ^]^ Aqueous batteries have been recognized as extremely promising candidates for large‐scale energy storage due to their environmental benignity, inherent safety, and ease of operation.^[^ ^2^ ^]^ In particular, aqueous zinc‐ion batteries (AZIBs) have attracted significant attention in aqueous battery development, owing to their superior chemical stability and elevated theoretical specific capacity.^[^ ^3^, ^4^, ^5^ ^]^ Nevertheless, the development of efficient cathode materials remains a major bottleneck, which limits the practical application in AZIBs.^[^ ^6^, ^7^ ^]^ Although some progress has been made in cathode material research, challenges such as the Jahn‐Teller effect in manganese‐based materials^[^ ^8^, ^9^ ^]^ and structural degradation in vanadium‐based materials^[^ ^10^, ^11^, ^12^ ^]^ still significantly impair battery performance. Hence, designing cathode materials that possess both high specific capacity and long‐term stability has become an urgent research hotspot and technical challenge in the AZIBs field.

Metal‐organic frameworks (MOFs) hold significant promise for energy storage applications owing to their high specific surface area, tunable pore structures, and diverse coordination environments.^[^ ^13^, ^14^, ^15^ ^]^ In particular, their porous architecture offers multiple pathways for ion migration and charge transport, which is beneficial for enhancing the electrochemical performance of energy storage materials.^[^ ^16^, ^17^, ^18^ ^]^ However, during the repeated charge‐discharge cycles, severe volume variations may induce the collapse of the framework structure, leading to rapid capacity degradation and a significant reduction in cycle life.^[^ ^19^ ^]^ To address the above problem, combining MOFs with other functional compounds has emerged as an effective strategy to improve MOFs’ electrochemical performance. Among them, polyoxometalates (POMs), due to their sub‐nanometer size and strong redox activity, are capable of reversibly accepting and releasing electrons without compromising structural integrity, making them ideal potential candidates.^[^ ^20^, ^21^ ^]^ Inspired by this, constructing novel host‐guest MOF@POM materials by confining electron‐rich POMs within porous MOFs may be an effective strategy to promote reversible redox reactions and enhance their electrochemical performance. From the perspective of morphological design, MOF@POM composites not only integrate the excellent ion transport channels of MOFs and the reversible charge transfer capabilities of POMs, but also expose more active sites due to their tailored porous structures.^[^ ^22^ ^]^ In terms of structural stability, the non‐covalent interactions between the MOF and POM further enhance the stability of the composite framework, allowing it to accommodate volume changes during charge/discharge cycles.^[^ ^23^, ^24^ ^]^ Due to the synergistic effect between MOFs and POMs, the MOF@POM composites is expected to exhibit better electrochemical performance in AZIBs than the pristine MOFs.

In this work, Br‐MIL(V)‐47 with two large pore diameters (13.93 and 16.34 Å) was selected as a template. This enabled the smaller‐sized Anderson‐type POM (FeMo_6_, 8.5∼9.5 Å) to be successfully confined within its pores and properly function. A series of spindle‐like MOF@POM materials Br‐MIL‐47@{(NH_4_)_3_[Fe(III)Mo_6_O_24_H_6_]·6H_2_O} (Br@P‐X) were prepared via a one‐step solution method. Driven by the synergistic effects of the multiple components and the accelerated migration of Zn^2+^ induced by POM, the novel cathode Br@P‐X exhibits superior electrochemical performance in AZIBs. Among them, the Br@P‐16 cathode retains a specific capacity of 95.6 mAh g^−1^ after 2500 cycles at 3 A g^−1^. Meanwhile, X‐ray absorption fine structure spectroscopy (XAFS) was employed to comprehensively analyze the coordination environment of vanadium centers, and in situ/ex situ characterization techniques were used to systematically investigate the structural evolution of the electrode material during the electrochemical cycling. Furthermore, the assembled flexible Zn/Br@P‐16 soft pack battery maintained a remarkable specific capacity of 88.7 mAh g^−1^ after 100 cycles at 1 A g^−1^, providing valuable insights for the future development of high‐performance and long‐lasting flexible batteries.

## 2 Results and Discussion

**Figure 1:**
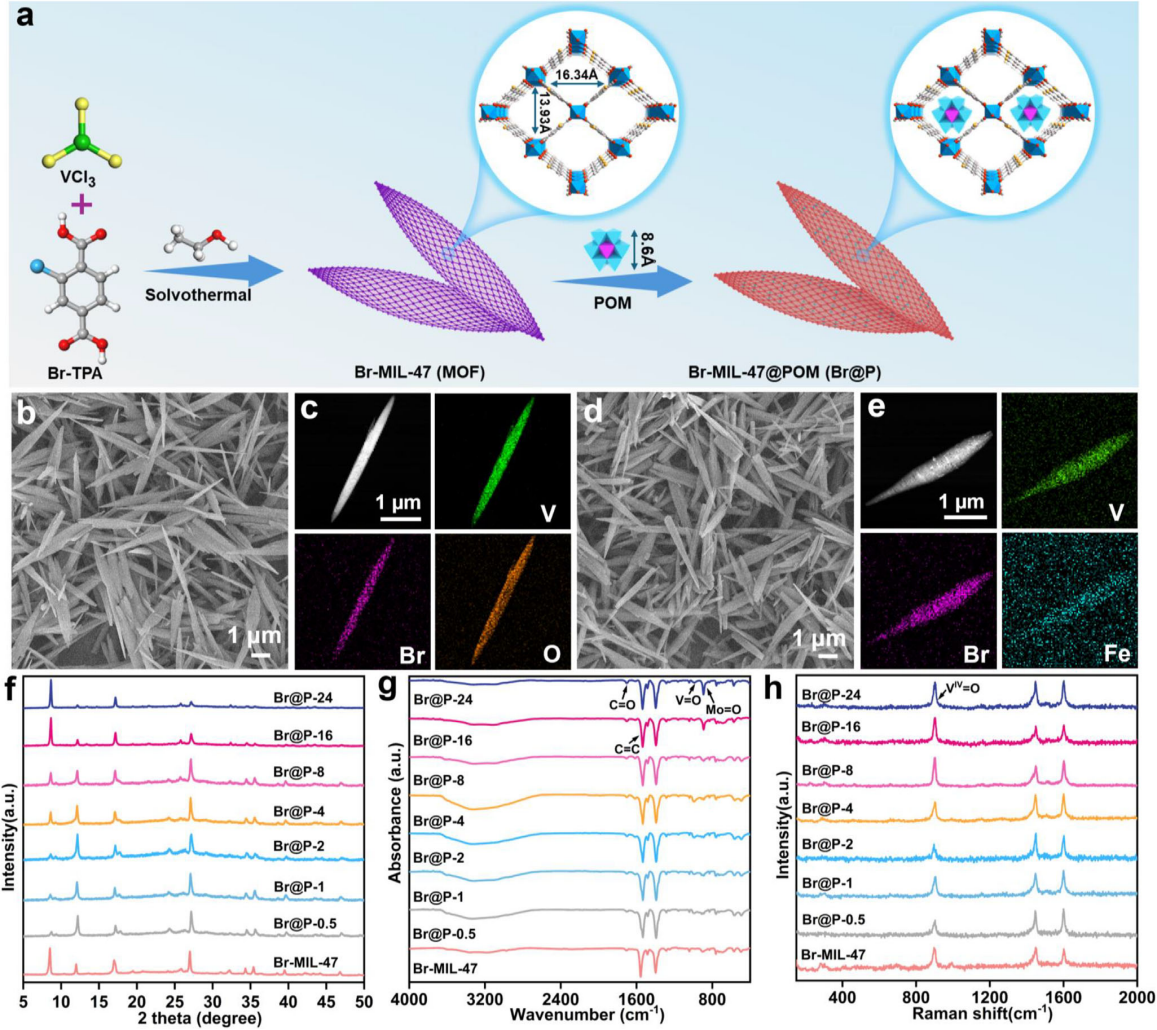
a) Schematic diagram of the Br@P‐X synthesis process. b) SEM images of Br‐MIL‐47. c) HAADF and EDX elemental mapping of Br‐MIL‐47. d) SEM images of Br@P‐16. e) HAADF and EDX elemental mapping of Br@P‐16. The comparison of f) XRD spectra, g) FTIR spectra, and h) Raman spectra of pure Br‐MIL‐47 and Br@P‐X.

Due to its unique mesoporous properties, Br‐MIL‐47 as an effective host for the encapsulation of Anderson‐type POMs. As depicted in **Figure** **1**a, FeMo_6_ was introduced into the precursor solution of Br‐MIL‐47 during the self‐assembly process to synthesize a series of hybrid materials, designated as Br@P‐X. Varying the stirring time allows precise control over FeMo_6_ loading, resulting in a series of materials ranging from Br@P‐0.5 to Br@P‐24. Scanning electron microscopy (SEM) images of Br@P‐X reveal a uniform spindle‐like structure with an average particle width of 0.51 µm, consistent with that of Br‐MIL‐47 (Figure 1b,d; Figures S1, S2, Supporting Information). The analysis of energy dispersive X‐ray (EDX) spectra and mapping images from energy dispersive spectroscopy (EDS) indicated a uniform distribution of V, O, C, Br, Fe, and Mo throughout the entire structure (Figure 1c,e; Figure S3, Supporting Information).

The crystalline phases of Br‐MIL‐47 and Br@P‐X were identified using powder X‐ray diffraction (XRD) as illustrated in Figure 1f. Br@P‐X exhibits diffraction patterns consistent with the crystal structure of Br‐MIL‐47, indicating that the introduction of FeMo_6_ barely affects the construction of its classical framework. This suggests that FeMo_6_ is successfully confined within the pores of Br‐MIL‐47, rather than merely adsorbed on its surface. The Fourier transform infrared (FTIR) spectrum depicted in Figure 1g exhibits an absorption at 1702 cm^−1^ for Br@P‐X, which is attributed to the stretching vibration ν(C═O) of the organic ligand. The peaks observed at 1552 and 1480 cm^−1^ correspond to the stretching vibrations ν(C═C) of the aromatic ring, whereas the peaks at 1400 and 1282 cm^−1^ are associated with the bending δ(O─H) and stretching vibrations ν(C─O), respectively. Moreover, the peak at 889 cm^−1^ is ascribed to the δ(Mo═O) bending vibrations in the {FeMo_6_O_24_} cluster.^[^ ^25^ ^]^ Raman spectroscopy was also performed to further verify the successful preparation of Br@P‐X (Figure 1h).

**Figure 2:**
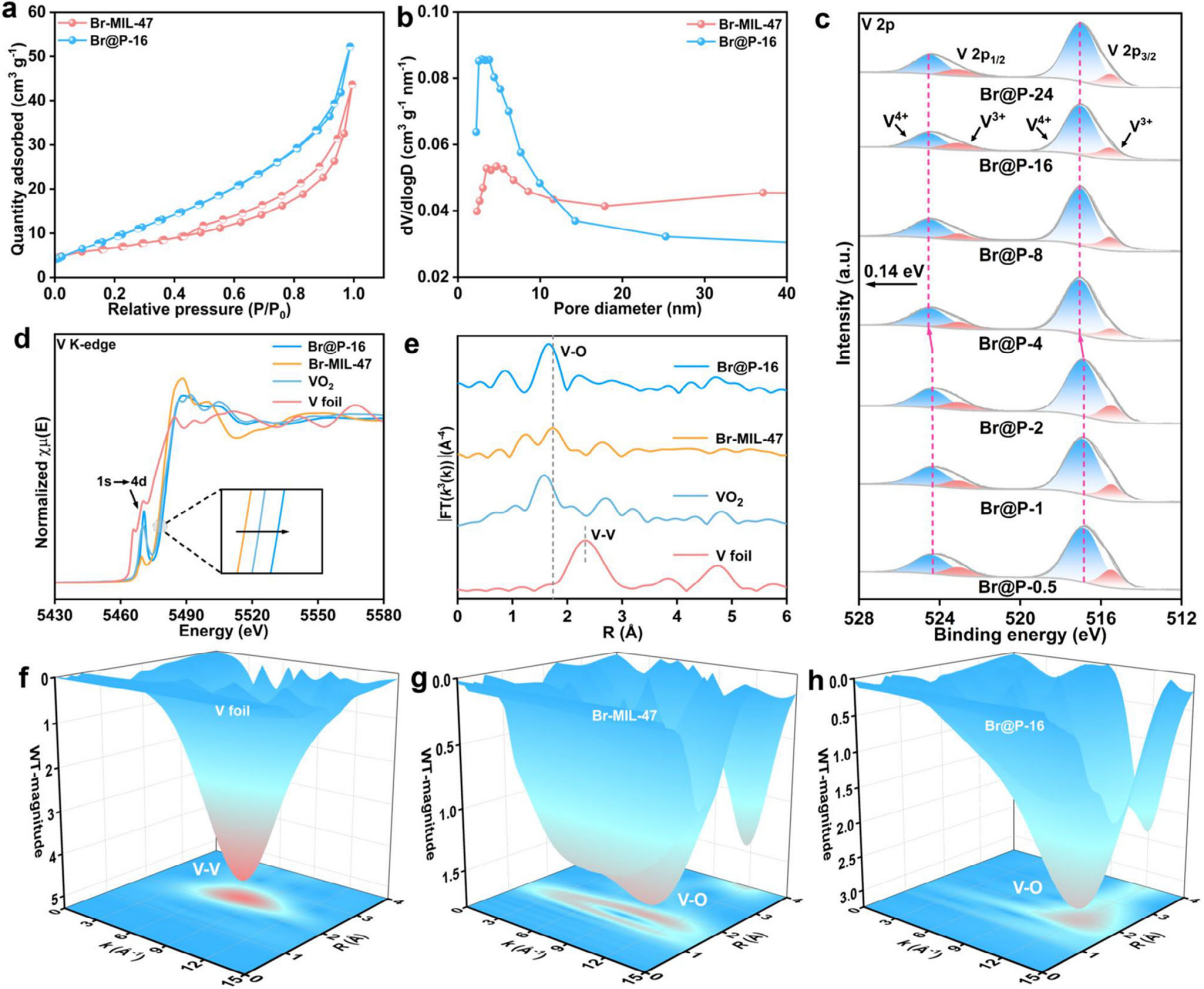
a) N_2_ adsorption‐desorption isotherms of Br‐MIL‐47 and Br@P‐16. b) Pore size distribution plots of Br‐MIL‐47 and Br@P‐16. c) V 2p XPS spectra of Br@P‐X. d) XANES spectra of V K‐edge. e) V K‐edge of Fourier‐transform EXAFS spectra. f,g,h) EXAFS wavelet transform Vfoil, Br‐MIL‐47, and Br@P‐16.

The porous characteristics of Br‐MIL‐47 and Br@P‐X materials were confirmed by N_2_ adsorption‐desorption measurements. The isotherms of Br‐MIL‐47 and Br@P‐X demonstrate typical Type IV behavior, characterized by a significant hysteresis loop, which suggests the existence of a mesoporous structure (**Figure** **2a**; Figure S4, Supporting Information).^[^ ^26^ ^]^ The specific surface areas of Br‐MIL‐47, Br@P‐0.5, Br@P‐1, Br@P‐2, Br@P‐4, Br@P‐8, Br@P‐16, and Br@P‐24 are 24.06, 27.50, 34.34, 36.84, 37.67, 38.98, 39.56, and 42.24 m^2^ g^−1^, respectively (Table S1, Supporting Information). This gradual increase in surface area can be primarily attributed to the uniform confinement of FeMo_6_ clusters within the pores of Br‐MIL‐47, which leads to an expansion of the pore structure. This confinement not only increases the surface area but also provides more active sites for Zn^2+^ adsorption and redox reactions, significantly enhancing the reversible specific capacity of the cathode. Meanwhile, as the FeMo_6_ loading increases, the average pore diameter decreases from 11.00 to 7.76 nm (Figure 2b; Figure S5, Supporting Information), indicating that FeMo_6_ clusters are primarily confined within the MOF framework. The reduced pore size shortens the ion diffusion pathway, which facilitates faster mass transport and improves the rate performance. Inductively coupled plasma optical emission spectrometry (ICP‐OES) was employed to analyze the V content in Br@P‐X and quantify the mass ratios among V, Fe, and Mo (Figure S6, Supporting Information).

X‐ray photoelectron spectroscopy (XPS) was utilized to examine the electronic configuration and coordination environment of Br@P‐X. The full XPS spectra reveal that the surface of the Br@P‐X sample is primarily composed of C, O, Br, V, Fe, and Mo, with no other significant impurities (Figure S7, Supporting Information), which is consistent with the elemental mapping results (Figure 1e). By deconvoluting the V 2p spectra of Br‐MIL‐47 and Br@P‐X (Figure 2c), two sets of peaks are observed, corresponding to the spin‐orbit split components of V^4+^ (516.98 eV for V 2p_3/2_ and 524.45 eV for V 2p_1/2_) and V^3+^ (515.52 eV for V 2p_3/2_ and 523.13 eV for V 2p_1/2_).^[^ ^27^ ^]^ With increasing FeMo_6_ loading, the V^4+^ peaks of Br@P‐X exhibit a noticeable shift toward higher binding energies compared to those of Br‐MIL‐47, indicating that the incorporation of FeMo_6_ significantly alters the electronic structure of Br@P‐X. Moreover, the peak area ratio of V^4+^ to V^3+^ in Br@P‐X is higher than that in Br‐MIL‐47, suggesting the formation of more V^4+^ active sites on the surface of Br@P‐X. The O 1s spectra of Br‐MIL‐47 and Br@P‐X (Figure S8a, Supporting Information) were deconvoluted into three peaks corresponding to O─H (533.55 eV), O = C (531.83 eV), and M─O (530.36 eV), respectively.^[^ ^28^ ^]^ The Fe 2p spectrum of Br@P‐X (Figure S8b, Supporting Information) shows three distinct peak pairs corresponding to Fe^3+^ (712.34 and 725.46 eV), Fe^2+^ (710.11 and 722.95 eV), and satellite features (716.79 and 729.99 eV), further confirming the successful confinement of FeMo_6_ within the Br@P‐X framework.

XAFS was employed to evaluate the electronic structure and the local atomic coordination of V centers in Br‐MIL‐47 and Br@P‐16. The V K‐edge X‐ray absorption near‐edge structure (XANES) spectra are shown in Figure 2d. The absorption‐edge energy positions of Br‐MIL‐47 and Br@P‐16 were higher than those of V foils and similar to those of VO_2_, indicating that the V oxidation states in Br‐MIL‐47 and Br@P‐16 were close to +4. Compared to Br‐MIL‐47, the V K‐edge XANES absorption edge of Br@P‐16 exhibited a positive shift, indicating that the incorporation of FeMo_6_ increased the average valence state of the V centers, accompanied by electron transfer and charge redistribution.^[^ ^29^, ^30^ ^]^ Meanwhile, the k^3^‐weighted Fourier‐transformed V K‐edge X‐ray absorption fine structure (EXAFS) spectra show two well‐defined peaks at approximately 1.66 and 2.33 Å (Figure 2e), which correspond to the apparent bond lengths of V─O and V─V, respectively. The slight shift of the V─O peak of Br@P‐16 compared to Br‐MIL‐47 is caused by local structural changes resulting from the successful confinement of FeMo_6_ within the pores of Br@P‐16. The corresponding wavelet transform diagrams in Figure 2f–h indicates that the strongest peaks in V foil originate from the V─V single scattering contribution.^[^ ^31^ ^]^ In contrast, the dominant peaks in Br‐MIL‐47 and Br@P‐16 are mainly attributed to V─O single scattering contribution, with the other peaks corresponding to the V─O scattering contribution.

**Figure 3:**
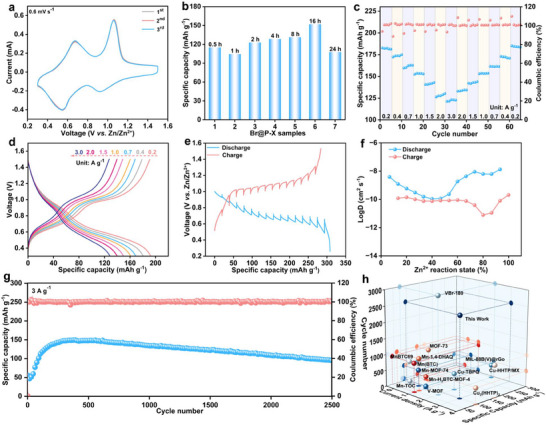
a) CV curves of Br@P‐16 cathode. b) Bar chart of cycle performance at 0.2 A g^−1^. c) Rate performance of Br@P‐16 cathode. d) GCD curves of Br@P‐16 cathode. e) GITT curve of Br@P‐16 cathode. f) Zn^2+^ diffusion coefficients (Log D_Zn_^2+^). g) Long‐term cyclability of Br@P‐16 cathode at 3 A g^−1^ for 2500 cycles. h) Comparison of electrochemical performance with recently reported cathodes.

Coin‐type Zn‐ion batteries were assembled using Br‐MIL‐47 as the cathode, Zn foil as the anode, and 3 m Zn(CF_3_SO_3_)_2_ solution as the electrolyte to evaluate their electrochemical characteristics. The Br@P‐X cathodes exhibit nearly overlapped cyclic voltammetry (CV) curves, indicating excellent redox reversibility (**Figure** **3**a; Figure S9, Supporting Information). Cycling stability plays a crucial role in assessing battery performance. Br@P‐16 exhibits a higher and more stable reversible specific capacity, reaching 151.9 mAh g^−1^ at the 100th cycle under a current density of 0.2 A g^−1^, which is significantly superior to those of pure FeMo_6_, pure Br‐MIL‐47, and other Br@P‐X cathodes (Figure 3b; Figures S10, S11, Supporting Information). To evaluate the rate performance of the Br@P‐X cathodes for rapid Zn^2+^ storage, galvanostatic tests were performed with current densities varying from 0.2 to 3.0 A g^−1^. As depicted in Figure 3c and Figure S12 (Supporting Information), Br@P‐16 exhibited the optimal rate performance among all tested cathodes. The average reversible capacities at 0.2, 0.4, 0.7, 1.0, 1.5, 2.0, and 3.0 A g^−1^ were 185.2, 172.5, 161.4, 153.6, 142.9, 134.4, and 123.2 mAh g^−1^, respectively. Moreover, when the current density was switched back to 0.2 A g^−1^, the capacity nearly recovered to its initial value, indicating excellent Zn^2+^ storage reversibility and structural stability. Figure 3d displays the galvanostatic charge/discharge (GCD) curves of the Br@P‐16 cathode at various current densities, all exhibiting well‐defined voltage plateaus, indicating good electrochemical reversibility and fast reaction kinetics.^[^ ^32^ ^]^

Electrochemical impedance spectroscopy (EIS) was utilized to evaluate the kinetics of charge transfer and Zn^2+^ transport in the Br@P‐X cathodes. Compared with Br@P‐0.5, Br@P‐1, Br@P‐2, Br@P‐4, Br@P‐8, and Br@P‐24, Br@P‐16 exhibits the lowest charge transfer resistance (220.7 Ω) in the Nyquist plot (Figures S13, S14, Supporting Information), with the corresponding equivalent circuit model shown in the insets. This indicates that FeMo_6_ is effectively confined within the pores of Br@P‐16, thereby facilitating interfacial charge transfer and enhancing Zn^2+^ diffusion kinetics.^[^ ^33^, ^34^ ^]^ The ion diffusion kinetics of the Br@P‐16 cathode was further investigated using the Zn^2+^ diffusion coefficient (D), which was calculated from the galvanostatic intermittent titration technique (GITT) measurements (Figure 3e). As illustrated in Figure 3f, the D value of Br@P‐16 cathode is in the range 10^−11^–10^−8^ cm^2^ s^−1^. Meanwhile, at 3.0 A g^−1^, the Br@P‐16 cathode delivers exceptional cycling stability, retaining a reversible capacity of 95.6 mAh g^−1^ even after 2500 cycles (Figure 3g), which reflects its outstanding electrochemical stability. Notably, the initial rise in specific capacity is attributed to the gradual activation of electroactive sites and improved electrode‐electrolyte wetting during early cycles, which facilitate enhanced Zn^2+^ accessibility and utilization.^[^ ^35^, ^36^ ^]^ SEM analysis (Figure S15, Supporting Information) reveals that, compared to the uncycled sample, the Br@P‐16 cathode maintains an overall intact framework after multiple charge‐discharge cycles, with only minor localized collapse observed, further confirming the excellent structural stability of the Br@P‐16 material during long‐term cycling. Therefore, based on the analysis of cycling performance and EIS spectra, the relatively low FeMo_6_ content in Br@P‐0.5 to Br@P‐8 was insufficient to effectively enhance electron conductivity and facilitate Zn^2+^ diffusion, resulting in lower reversible specific capacities. In contrast, the excessive FeMo_6_ loading in Br@P‐24 not only introduced impurities and reduced the proportion of electroactive metal‐organic framework components, but also increased the charge transfer resistance of the electrode, thereby deteriorating its electrochemical performance. These results indicate that the electrical conductivity and Zn^2+^ transport behavior of the cathode materials could be effectively regulated by precisely tuning the FeMo_6_ loading in Br@P‐X, thus achieving optimal Zn storage performance.^[^ ^24^ ^]^ As depicted in Figure 3h and Table S2 (Supporting Information), the Br@P‐16 cathode demonstrates superior electrochemical performance compared to most reported aqueous cathode materials.

**Figure 4:**
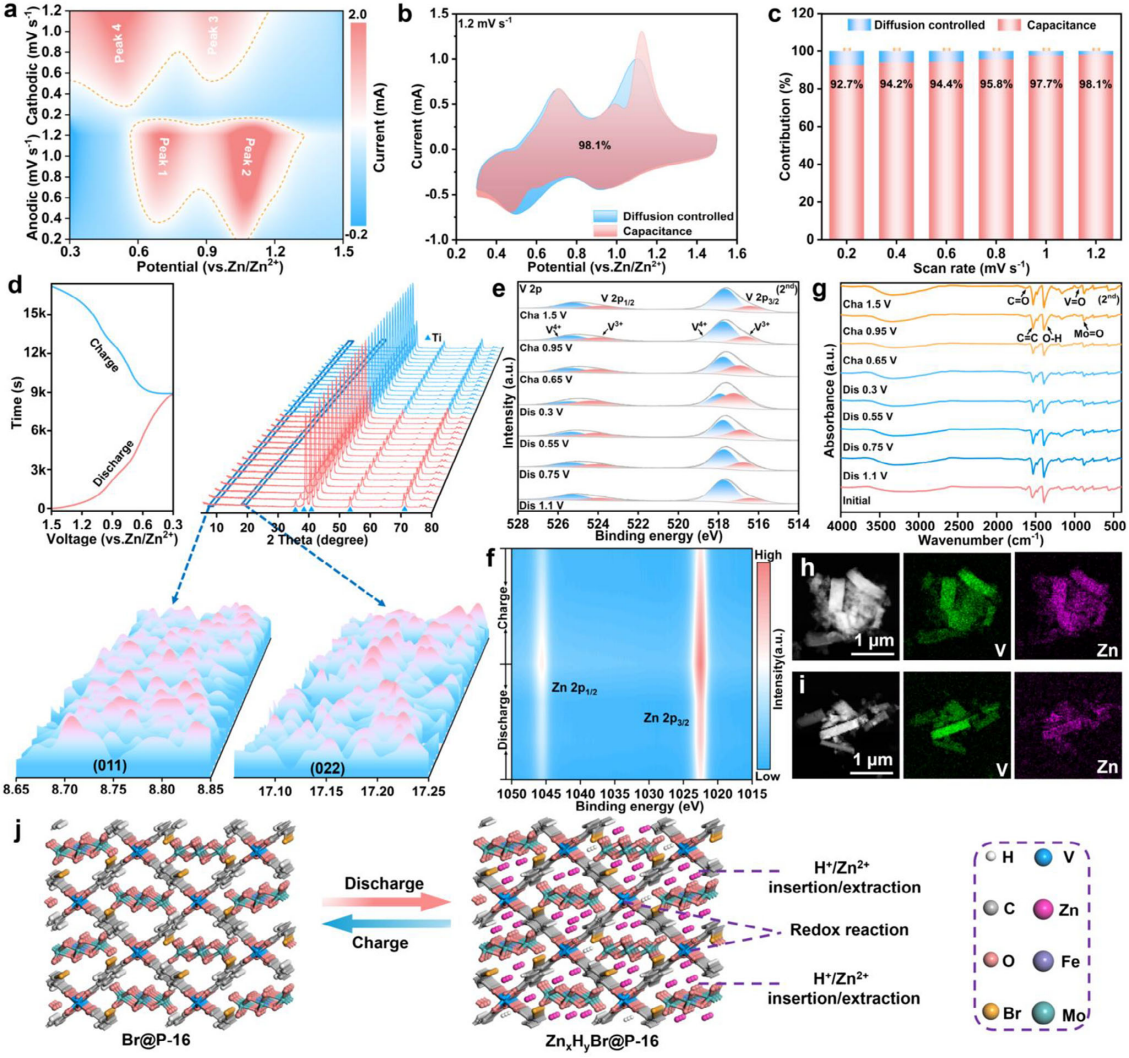
a) Contour plots of CV patterns for Br@P‐16. b) The capacitive contribution for Br@P‐16 cathode at 1.2 mV s^−1^. c) Capacitive contributions of Br@P‐16 cathode. d) In situ XRD characterization of Br@P‐16 cathode. e) V 2p, f) Zn 2p XPS spectra of Br@P‐16 cathode at various states. g) Ex situ FTIR spectra of Br@P‐16 cathode. HAADF‐STEM and EDX elemental mapping of the Br@P‐16 cathode at (h) discharge 0.3 V and (i) charge 1.5 V. j) Schematic illustration of the charge‐discharge mechanism of the Br@P‐16 cathode.

To explore the electrochemical kinetics of Br@P‐X cathodes, CV curves were recorded at scan rates ranging from 0.2 to 1.2 mV s^−1^ (**Figure** **4**a; Figure S16, Supporting Information). All Br@P‐X cathodes exhibit two distinct pairs of prominent redox peaks in their CV curves, indicating a multistep (de)intercalation reaction involving Zn^2+^ and H^+^. For the Br@P‐16 cathode, the CV curves retained similar shapes as the scan rate increased, while their peak areas gradually enlarged. As a result of polarization effects, the oxidation and reduction peaks exhibited positive and negative potential shifts, respectively.^[^ ^37^, ^38^ ^]^ The current responses of Br@P‐X materials were fitted using the pseudocapacitive contribution formula provided in the Supporting Information, and the corresponding b‐values were obtained. According to the fitting parameter b (see Supporting Information for details), the b‐values of the Br@P‐16 cathode are 0.989 and 0.799 for the two oxidation peaks, and 0.984 and 0.881 for the corresponding reduction peaks (Figure S17, Supporting Information), indicating that the electrochemical process involves both capacitive and diffusion‐controlled contributions, with capacitive behavior being dominant.^[^ ^39^ ^]^ Further analysis revealed that the capacitive contributions of Br@P‐16 at different scan rates are 92.7%, 94.2%, 94.4%, 95.8%, 97.7%, and 98.1%, all of which are higher than those of other Br@P‐X cathodes. The high capacitive contribution reflects the excellent kinetic properties of Br@P‐16, thereby imparting exceptional rate performance and cycling stability (Figure 4b,c; Figures S18–S25, Supporting Information).^[^ ^40^, ^41^ ^]^

To reveal the reaction mechanism and structural evolution of Br@P‐16 during cycling, in situ and ex situ analyses were performed. Figure 4d presents the electrochemical behavior of the Br@P‐16 cathode during a typical GCD process and its corresponding in situ XRD pattern. During the discharge process, the diffraction peaks of the (011) and (022) planes, located at ≈ 8.7° and 17.1°, gradually shifted to lower angles, indicating the insertion of Zn^2+^/H^+^ ions into the Br@P‐16 crystal structure, resulting in lattice expansion.^[^ ^42^ ^]^ In contrast, during the charging process, as the voltage increased to 1.5 V, the diffraction peaks of the (011) and (022) planes gradually returned to their original positions, suggesting that the Br@P‐16 cathode exhibits good structural reversibility during Zn^2+^/H^+^ insertion/extraction.^[^ ^43^ ^]^

Ex situ XPS was used to examine the changes of chemical composition and elemental valence of Br@P‐16 cathode in the second cycle (Figure S26, Supporting Information). The V 2p XPS spectrum (Figure 4e) clearly reveals the presence of V^4+^ and V^3+^ species. Upon discharge to 0.3 V, the intensity of the V^4+^ peak decreases, while that of the V^3+^ peak increases significantly, indicating the reduction of V^4+^ to V^3+^ due to Zn^2+^ insertion.^[^ ^44^, ^45^ ^]^ When the Br@P‐16 cathode is charged to 1.5 V, the V^4+^ peak becomes dominant and the V^3+^ component decreases. The V 2p spectrum almost returns to its initial state, indicating that a highly reversible redox reaction of V species occurs. And the Zn 2p spectrum shown in Figure 4f. During discharge, two characteristic peaks at approximately 1022.1 and 1045.6 eV correspond to Zn 2p_3/2_ and Zn 2p_1/2_, respectively, indicating the insertion of Zn^2+^ into the Br@P‐16 cathode. When charged to 1.5 V, the signal intensity of the Zn 2p peak decreases, indicating the reversible extraction of Zn^2+^.^[^ ^46^ ^]^ Subsequently, ex situ FTIR spectra provided additional insights into the structural dynamics of the Br@P‐16 cathode (Figure 4g). Compared with the initial state, the characteristic absorption peaks located at ≈1670–1290 cm^−1^ and 1020–800 cm^−1^ gradually change in intensity and exhibit slight shifts during the discharge process (from 1.1 to 0.3 V), indicating that the Mo═O and V═O coordination bonds are involved in the reduction reaction. During the charging process (from 0.65 to 1.5 V), these peaks gradually return to their original state, further confirming the excellent reversibility of the oxygen‐containing coordination groups. It is worth noting that during the discharge process, the vanadium centers undergo a reduction from V^4+^ to V^3+^, as confirmed by XPS and CV analyses. Considering the multi‐electron redox capability of the FeMo_6_ cluster, part of the introduced electrons may further migrate and delocalize onto the FeMo_6_ clusters, thereby alleviating local electron accumulation at the V sites, relieving structural stress, and enhancing cycling stability. During the charging process, the electrons can reversibly transfer back from the FeMo_6_ cluster to the V sites, forming a reversible redox cycle. Such interfacial charge redistribution mechanisms have also been reported in other MOF@POM systems and are commonly associated with improved electrochemical reversibility.^[^ ^47^, ^48^ ^]^ Furthermore, the EDX elemental mapping of the Br@P‐16 cathode at different charge/discharge states further confirms the reversible insertion/extraction of Zn^2+^ (Figure 4h,i; Figures S27–S29, Supporting Information), which is consistent with the ex situ XPS analysis. Accordingly, the energy storage mechanism of the Br@P‐16 cathode is presented in Figure 4g.

**Figure 5:**
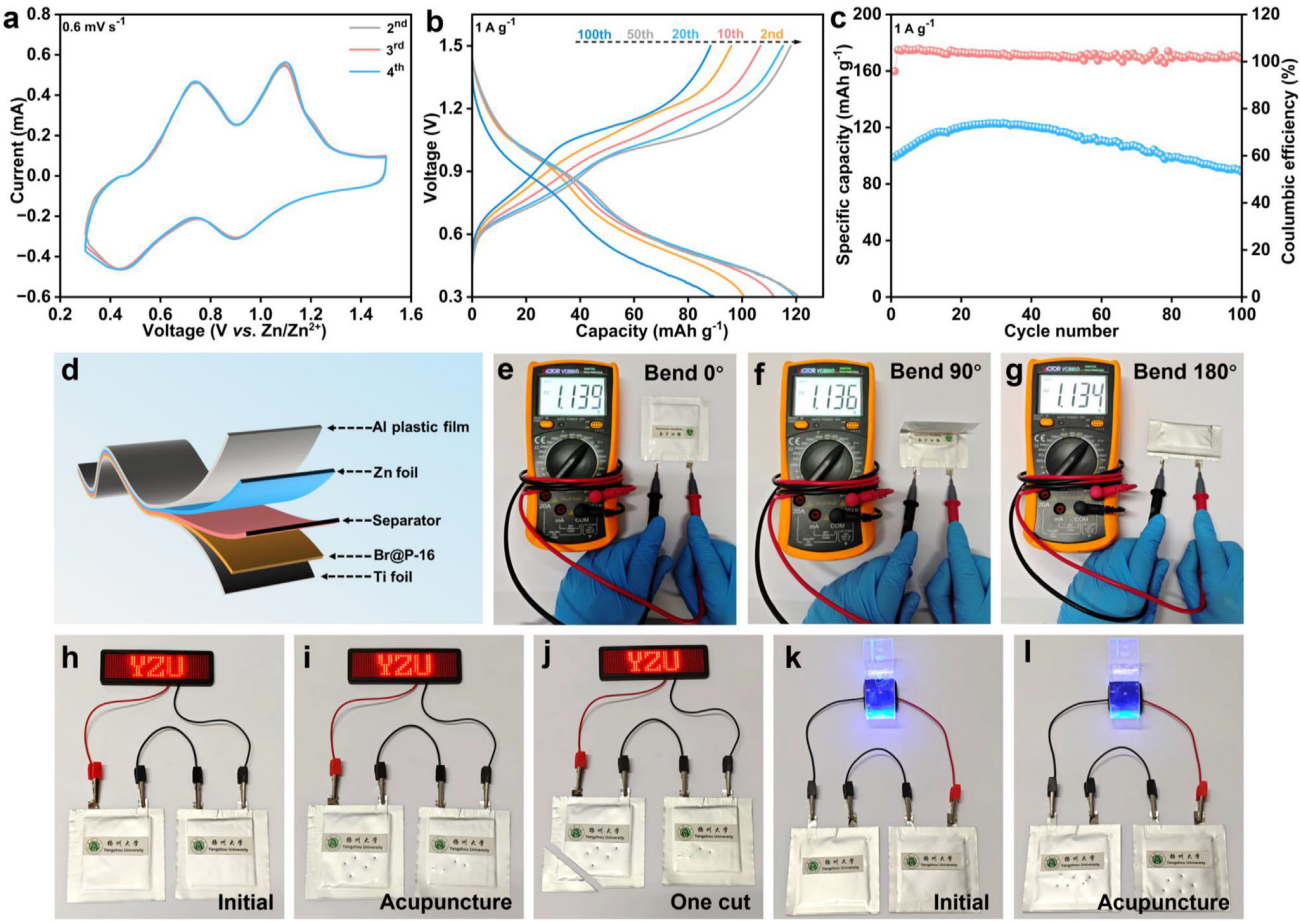
Electrochemical performance of Zn/Br@P‐16 soft pack battery. a) CV curves. b) GCD curves. c) Long cycle performance at 1A g^−1^. d) Schematic diagram of soft pack battery. Open‐circuit voltage under (e) 0°, (f) 90°, and (g) 180° bending states. Light up the LED sign in the (h) initial, (i) acupuncture, and (j) one‐cut states. Powering the wristband in the (k) initial and (l) acupuncture states.

In recent years, flexible energy storage devices have attracted significant interest due to their high energy density, lightweight design, and flexibility.^[^ ^49^, ^50^ ^]^ The outstanding electrochemical characteristics of the Br@P‐16 cathode material has prompted us to further explore its practical applications. The CV curves of the Zn/Br@P‐16 soft pack battery exhibit two pairs of redox peaks (**Figure** **5**a), which are consistent in shape and position with those observed in the coin cell (Figure 3a), indicating a reversible Zn^2+^/H^+^ insertion/extraction process in Br@P‐16. The GCD curves of the Zn/Br@P‐16 soft pack battery demonstrate excellent reversibility and stability throughout the electrochemical process (Figure 5b). As presented in Figure 5c, the Zn/Br@P‐16 soft pack battery delivers a specific capacity of 88.7 mAh g^−1^ after 100 cycles. Figure 5d illustrates the assembly schematic of the Zn/Br@P‐16 soft‐pack battery, comprising Br@P‐16 cathode, zinc foil anode, and glass fiber separator. Under various bending states, the open‐circuit voltage remained consistently stable at ≈1.13 V for a single Zn/Br@P‐16 soft‐pack battery and ≈2.35 V for two devices connected in series (Figure 5e–g; Figures S30, S31, Supporting Information), demonstrating excellent mechanical stability. Moreover, two Zn/Br@P‐16 soft pack batteries connected in series successfully powered an LED sign and a wristband, achieving sustained and stable device operation (Figure 5h–l; Figure S32, Supporting Information), which demonstrates the excellent safety and operational stability of the battery in practical applications.

## 3 Conclusion

In summary, a facile one‐step solution method was applied to achieve the efficient confinement of POMs within MOF pores. A series of novel Br@P‐X hybrid nanomaterials were successfully synthesized and served as cathode materials for AZIBs. By virtue of the reversible multi‐electron redox activity of FeMo_6_ and the porous architecture of Br‐MIL‐47, a high specific capacity was realized. Meanwhile, the non‐covalent interactions between FeMo_6_ and Br‐MIL‐47 significantly improve the electronic conductivity and structural stability of the hybrid nanomaterials. Consequently, the optimized Br@P‐16 cathode delivers 95.6 mAh g^−1^ after 2500 cycles at 3 A g^−1^ and exhibits outstanding reversibility, rate performance, and cycling stability. In situ XRD and ex situ XPS/FTIR analyses demonstrated the dynamic structural transformation of Br@P‐16 and confirmed its highly reversible Zn^2+^ storage mechanism. Moreover, the assembled flexible Zn/Br@P‐16 soft pack battery continuously powered an LED sign and a wristband, demonstrating excellent potential for practical application and offering new insights for the design of next‐generation AZIBs.

## Conflict of Interest

The authors declare no conflict of interest.

## Supporting information



Supporting Information

Supporting Information

## Data Availability

The data that support the findings of this study are available from the corresponding author upon reasonable request.

## References

[1] Z. X. Liu , Y. Huang , Y. Huang , Q. Yang , X. Li , Z. Huang , C. Zhi , Chem. Soc. Rev. 2020, 49, 180. 31781706 10.1039/c9cs00131j

[2] W. Zhang , G. He , Angew. Chem., Int. Ed. 2023, 62, 202218466.

[3] L. Geng , J. Meng , X. Wang , W. Wu , K. Han , M. Huang , L. Mai , Chem 2025, 11, 102302.

[4] W. Liu , Q. Zhao , S. He , H. Yu , Y. Li , P. Cao , G. Kuang , B. Xu , Y. Chen , L. Chen , Adv. Funct. Mater. 2025, 10.1002/adfm.202425680.

[5] F. Wan , Z. Hao , S. Wang , Y. Ni , J. Zhu , Z. Tie , S. Bi , Z. Niu , J. Chen , Adv. Mater. 2021, 33, 2102701. 10.1002/adma.202102701 34302405

[6] Y. Zhao , S. Zhang , Y. Zhang , J. Liang , L. Ren , H. Fan , X. Sun , Energy Environ. Sci. 2024, 17, 1279.

[7] Y. Zhang , Q. Li , W. Feng , H. Yue , S. Gao , Y. Su , H. Pang , Angew. Chem., Int. Ed. 2025, 64, 202501728. 10.1002/anie.202501728 40017316

[8] X. Zhu , F. Meng , Q. Zhang , L. Xue , H. Zhu , S. Lan , H. Xia , Nat. Sustain. 2021, 4, 392.

[9] X. Wang , Q. Zhang , C. Zhao , H. Li , B. Zhang , G. Zeng , S. Sun , Nat. Energy 2024, 9, 184.

[10] T. Wang , P. Wang , L. Pan , Z. He , L. Dai , L. Wang , S. Liu , S. Jun , B. Lu , S. Liang , J. Zhou , Adv. Energy. Mater. 2023, 13, 2203523.

[11] J. Zhang , W. Zhou , D. Zhao , Y. Lu , D. Chao , Nat. Rev. Electr. Eng. 2025, 2, 215.

[12] M. J. Chen , S. Y. Tian , Y. X. Song , B. A. Lu , Y. Tang , J. Zhou , J. Cent. South Univ. 2024, 31, 4549.

[13] Y. Song , P. Ruan , C. Mao , Y. Chang , L. Wang , L. Dai , P. Zhou , B. Lu , J. Zhou , Z. He , Nano‐Micro Lett. 2022, 14, 218. 10.1007/s40820-022-00960-z PMC9646683 36352159

[14] G. Chen , C. Chen , Y. Guo , Z. Chu , Y. Pan , G. Liu , G. Liu , Y. Han , W. Jin , N. Xu , Science 2023, 381, 1350. 37733840 10.1126/science.adi1545

[15] L. Zou , C. Hou , Z. Liu , H. Pang , Q. Xu , J. Am. Chem. Soc. 2018, 140, 15393. 30347154 10.1021/jacs.8b09092

[16] N. Sun , S. Shah , Z. Lin , Y. Zheng , L. Jiao , H. Jiang , Chem. Rev. 2025, 125, 2703. 40070208 10.1021/acs.chemrev.4c00664

[17] P. Geng , L. Wang , M. Du , Y. Bai , W. Li , Y. Liu , S. Chen , P. Braunstein , Q. Xu , H. Pang , Adv. Mater. 2022, 34, 2107836. 10.1002/adma.202107836 34719819

[18] W. Li , X. Guo , P. Geng , M. Du , Q. Jing , X. Chen , G. Zhang , H. Li , Q. Xu , P. Braunstein , H. Pang , Adv. Mater. 2021, 33, 2107836. 10.1002/adma.202105163 34554610

[19] Y. Wu , Y. Bu , X. Li , X. Dong , X. Zhou , Z. Bu , Y. Wu , Adv. Funct. Mater. 2025, 10.1002/adfm.202500197.

[20] J. Lei , X. Fan , T. Liu , P. Xu , Q. Hou , K. Li , J. Chen , Nat. Commun. 2022, 13, 202. 35017484 10.1038/s41467-021-27866-5 PMC8752791

[21] H. Wang , S. Hamanaka , Y. Nishimoto , S. Irle , T. Yokoyama , H. Yoshikawa , K. Awaga , J. Am. Chem. Soc. 2012, 134, 4918. 22352694 10.1021/ja2117206

[22] Y. Liu , X. Zhou , T. Qiu , R. Yao , F. Yu , T. Song , X. Lang , Q. Jiang , H. Tan , Y. Li , Y. Li , Adv. Mater. 2024, 36, 2407705. 10.1002/adma.202407705 38925587

[23] Y. Liang , H. Zhang , M. Huo , X. Zhang , K. Qin , H. Wang , G. Zhu , Adv. Mater. 2025, 37, 2415545. 10.1002/adma.202415545 39711259

[24] G. Yuan , H. Ge , W. Shi , J. Liu , Y. Zhang , X. Wang , Angew. Chem., Int. Ed. 2023, 62, 202309934. 10.1002/anie.202309934 37551751

[25] Y. Liang , H. Jiang , H. Lin , C. Wang , K. Yu , C. Wang , J. Lv , B. Zhou , Chem. Eng. J. 2023, 466, 143220.

[26] X. Li , L. Yang , Q. Liu , W. Bai , H. Li , M. Wang , Q. Qian , Q. Yang , C. Xiao , Y. Xie , Adv. Mater. 2023, 35, 2304532. 10.1002/adma.202304532 37595959

[27] Q. Zong , Q. Wang , C. Liu , D. Tao , J. Wang , J. Zhang , H. Du , J. Chen , Q. Zhang , G. Cao , ACS Nano 2022, 16, 4588. 35258924 10.1021/acsnano.1c11169

[28] Y. Zheng , C. Tian , Y. Wu , L. Li , Y. Tao , L. Liang , G. Yu , J. Sun , S. Wu , F. Wang , Y. Pang , Z. Shen , Z. Pan , H. Chen , J. Wang , Energy Storage Mater. 2022, 52, 664.

[29] T. Lv , G. Zhu , S. Dong , Q. Kong , Y. Peng , S. Jiang , G. Zhang , Z. Yang , S. Yang , X. Dong , H. Pang , Y. Zhang , Angew. Chem., Int. Ed. 2023, 62, 202216089. 10.1002/anie.202216089 36409041

[30] Y. Su , Y. Zhang , W. Feng , G. Zhang , Y. Sun , C. Yin , G. Yuan , Y. Tang , W. Zhou , H. Chen , H. Pang , Angew. Chem., Int. Ed. 2025, 64, 202502752. 10.1002/anie.202502752 40088192

[31] K. Zhu , S. Wei , H. Shou , F. Shen , S. Chen , P. Zhang , C. Wang , Y. Cao , X. Guo , M. Luo , H. Zhang , B. Ye , X. Wu , L. He , L. Song , Nat. Commun. 2021, 12, 6878. 34824249 10.1038/s41467-021-27203-w PMC8617200

[32] Y. F. Zhang , Q. Li , W. C. Feng , S. J. Gao , H. T. Yue , Y. C. Su , H. J. Zhou , J. F. Huang , L. F. Han , M. Shakouri , Y. G. Wang , H. Pang , Adv. Mater. 2025, 10.1002/adma.202507609. 40495710

[33] W. Fan , S. Tian , L. Qin , T. Alomar , P. Ruan , Z. El‐Bahy , N. AlMasoud , B. Lu , J. Zhou , J. Am. Chem. Soc. 2025, 147, 18694. 40397793 10.1021/jacs.5c01648

[34] W. Liu , Q. Zhao , R. Jiang , X. Ni , T. You , C. Li , Y. Deng , B. Xu , Y. Chen , L. Chen , Adv. Powder Mater. 2025, 4, 100276.

[35] Y. Ran , M. Li , H. Zhao , J. Ren , Y. Sheng , G. Shao , Y. Wang , Y. Lei , Adv. Funct. Mater. 2025, 2510241.

[36] S. Wang , X. Guo , K. Huang , A. Achari , J. Safaei , Y. Lei , D. Li , Q. Gu , C. Sun , L. Gloag , S. Langford , A. Geim , R. R. Nair , G. Wang , Nat. Commun. 2025, 16, 5191. 40467665 10.1038/s41467-025-60558-y PMC12137938

[37] Y. Liu , X. Zhou , T. Qiu , R. Yao , F. Yu , T. Song , X. Lang , Q. Jiang , H. Tan , Y. Li , Adv. Mater. 2024, 36, 2407705. 10.1002/adma.202407705 38925587

[38] L. Xing , C. Zhang , M. Li , P. Hu , X. Zhang , Y. Dai , X. Pan , W. Sun , S. Li , J. Xue , Q. An , L. Mai , Energy Storage Mater. 2022, 52, 291.

[39] D. Zhao , X. Wang , W. Zhang , Y. Zhang , Y. Lei , X. Huang , Q. Zhu , J. Liu , Adv. Funct. Mater. 2023, 33, 2211412.

[40] Q. Li , Y. Zhang , X. Guo , Z. Yang , Y. Wang , Y. Chen , Y. Liu , H. Yue , S. Gao , H. Zhou , J. Huang , M. Shakouri , Y. Wang , G. Zhu , Z. Liu , Y. Zhang , H. Pang , Angew. Chem., Int. Ed. 2025, 64, 202509741. 10.1002/anie.202509741 40406804

[41] X. T. Guo , H. Y. Xu , Y. J. Tang , Z. B. Yang , F. Dou , W. T. Li , Q. Li , H. Pang , Adv. Mater. 2024, 36, 2408317. 10.1002/adma.202408317 39081106

[42] X. Wang , Y. Wang , A. Naveed , G. Li , H. Zhang , Y. Zhou , A. Dou , M. Su , Y. Liu , R. Guo , C. Li , Adv. Funct. Mater. 2023, 33, 2306205.

[43] D. Jia , Z. Shen , Y. Lv , Z. Chen , H. Li , Y. Yu , J. Qiu , X. He , Adv. Funct. Mater. 2023, 34, 2308319.

[44] L. Zhang , Y. Han , Y. Geng , H. Zhang , H. Liu , Y. He , Z. Yan , Z. Zhu , Angew. Chem., Int. Ed. 2025, 64, 202500434. 10.1002/anie.202500434 39985202

[45] Y. Geng , W. Xin , L. Zhang , Y. Han , H. Peng , M. Yang , H. Zhang , X. Xiao , J. Li , Z. Yan , Z. Zhu , F. Cheng , Nat. Sci. Rev. 2025, 12, nwae397. 10.1093/nsr/nwae397 PMC11740509 39831003

[46] X. Hu , Z. He , Q. Zhao , J. Zhou , C. Wang , S. Huang , G. Zhou , B. Xu , B. Wang , L. Chen , Y. Chen , Adv. Funct. Mater. 2024, 34, 2409247.

[47] K. Yue , R. Lu , M. Gao , F. Song , Y. Dai , C. Xia , B. Mei , H. Dong , R. Qi , D, Z. , J. Zhang , Z. Wang , F. Huang , B. Xia , Y. Yan , Science 2025, 388, 430. 40273253 10.1126/science.ads1466

[48] W. Ahmad , N. Ahmad , K. Wang , S. Aftab , Y. Hou , Z. Wan , B. Yan , Z. Pan , H. Gao , C. Peung , Y. Junke , C. Liang , Z. Lu , W. Yan , M. Ling , Adv. Sci. 2024, 11, 2304120. 10.1002/advs.202304120 PMC10837383 38030565

[49] S. Zhao , Y. Zuo , T. Liu , S. Zhai , Y. Dai , Z. Guo , Y. Wang , Q. He , L. Xia , Y. Zhi , J. Bae , K. Wang , M. Ni , Adv. Energy Mater. 2021, 11, 2101749.

[50] S. Deng , B. Xu , J. Zhao , C. Kan , X. Liu , Angew. Chem., Int. Ed. 2024, 63, 202401996. 10.1002/anie.202401996 38445364

