# Supplementary material for: Host‐Guest Synergistic Regulation in Functionalized Metal‐Organic Frameworks for Efficient Aqueous Zinc‐Ion Batteries
Source: Adv Sci (Weinh). 2025 Aug 11;12(42):e11198. doi: 10.1002/advs.202511198 (PMC12622505; doi:10.1002/advs.202511198)
Supplement: Supplementary file 1 — Supporting Information [file ADVS-12-e11198-s001.docx]

**Host-Guest Synergistic Regulation in Functionalized Metal-Organic Frameworks for Efficient Aqueous Zinc-Ion Batteries**

*Yanfei Zhang, Qian Li, Yichun Su, Yangyang Sun, Shuai Cao, Shengjie Gao, Haotian Yue,* *Hsiao-Chien Chen, and Huan Pang**

Y. Zhang, Q. Li, Y. Su, Y. Sun, S. Cao, S. Gao, H. Yue, H. Pang

School of Chemistry and Chemical Engineering, Yangzhou University, Yangzhou, 225002 Jiangsu, P. R. China.

S. Gao

College of Chemistry and Chemical Engineering, Chongqing University of Science and Technology, Chongqing, 401331, P.R. China.

H. Yue

School of Environmental Science, Nanjing Xiaozhuang University, Nanjing, Jiangsu, 211171, P.R. China.

H. Chen

Center for Reliability Science and Technologies, Chang Gung University, Kidney Research Center, Department of Nephrology, Chang Gung Memorial Hospital, Linkou, Taoyuan 333, Taiwan.

* Corresponding author.

*E-mail addresses*: panghuan@yzu.edu.cn, huanpangchem@hotmail.com (H. Pang)

**1. Experimental section**

**1.1 Chemical reagent**

All reagents were of analytical grade and could be used without further purification. Vanadium chloride (VCl_3_, 98%), 2-Bromoterephthalic acid (C_8_H_5_BrO_4_, 98%), Ferric sulfate (Fe_2_(SO_4_)_2_, 99.95%), Zinc trifluoromethanesulfonate ((CF_3_SO_3_)_2_Zn, 98%) were purchased from Aladdin. Ammonium molybdate tetrahydrate (H_24_Mo_7_N_6_O_24_·4H_2_O, 99%) was purchased from Sinopharm chemical reagent Co., Ltd. Ethanol (C_2_H_6_O, 99.7%) was purchased from Sinopharm chemical reagent Co., Ltd. N-Methyl-2-pyrrolidone (C_5_H_9_NO, 98%) and polyvinylidene fluoride were purchased from Meryer. All aqueous solutions were prepared with high-purity de-ionized water (DI water, resistance 18 MΩ cm^-1^).

## **1.2 Instrument required for experiment**

Electronic analytical balance (GL224-1SCN), purchased from Sartorius Scientific Instruments (Beijing) Co., Ltd. The phase and crystal structure of the material were characterized by X-ray diffraction (XRD) on a Bruker D8 Advanced X-ray Diffractometer (Cu-Kα radiation: λ = 0.15406 nm). The morphology of samples was observed by scanning electron microscope (SEM, Zeiss_Supra55) under the acceleration voltage of 5.0 kV. Transmission electron microscopy (TEM) investigations were performed by a JEM-2100 instrument. Energy dispersive X-ray spectrometry (EDX) elemental mapping scans were recorded using Tecnai G2 F30 S-TWIN at an acceleration voltage of 300 kV. The chemical states were measured using an Axis Ultra X-ray photoelectron spectroscope (XPS, Thermo Fisher Scientific ESCALAB250Xi) equipped with a standard monochromatic Al-Kα source (hv = 1486.6 eV). Fourier transform infrared (FTIR) transmission spectra were obtained on a BRUKER-EQUINOX-55 IR spectrophotometer. The products were tested by Raman on a DXRxi Raman Imaging Microscope for functional group analysis. Autosorb-Iq obtained the N_2_ sorption isothermals and pore size distribution *via* Brunauer-Emmet-Teller (BET) method. ICP-OES measurements were performed using a simultaneous ICP spectrometer (model Optima 7300 DV; PerkinElmer Inc., USA) equipped with a solid-state detector. The X-ray absorption fine structure spectroscopy (XAFS) measurements were performed at the Spring-8 (Japan) 12B2 Taiwan beamline of the National Synchrotron Radiation Research Center (NSRRC, Hsinchu, Taiwan). The XAFS datas were background subtracted from the overall absorption, normalized, and Fourier transformed by standard procedures within the Athena program.^[1]^ Electrochemical workstation (CHI-760E), purchased from CH Instruments.

**1.3 Materials synthesis**

**Synthesis of (NH_4_)_3_[Fe(III)Mo_6_O_24_H_6_]·6H_2_O (FeMo_6_):**

In a typical synthesis, Fe_2_(SO_4_)_3_·6H_2_O (1.2 g, 3.1 mmol) was dissolved in 20 mL of deionized water and slowly added dropwise into a boiling aqueous solution of (NH_4_)_6_Mo_7_O_24_·4H_2_O (5.2 g, 4.2 mmol) in 80 mL of deionized water under continuous stirring at 400 rpm. The solution obtained further evaporated on a steam bath and cooled to room temperature to yield the yellow crystals of FeMo_6_. The product was collected by filtration and dried at 50 °C for 24 h.

**Synthesis of Br-MIL-47(V):**

VCl_3_ (0.315 g, 2 mmol) and C_8_H_5_BrO_4_ (0.490 g, 2 mmol) were added to 10 mL of ethanol and stirred at 550 rpm for 30 min, followed by ultrasonic treatment at 40 kHz for 15 min. The mixture was then transferred to an autoclave and heated at 180 °C for 48 h. After naturally cooling to room temperature, the precipitate was separated by centrifugation at 8000 rpm, washed three times with ethanol, and finally dried under vacuum at 60 °C for 12 h.

**Synthesis of Br-MIL-47@POM (Br@P-X):**

FeMo_6_ (0.1 mM) was dispersed in 50 mL of an ethanol solution containing Br-MIL-47 (100 mg), and the mixture was stirred for 0.5, 1, 2, 4, 8, 16, and 24 h to obtain Br@P-0.5, Br@P-1, Br@P-2, Br@P-4, Br@P-8, Br@P-16, and Br@P-24, respectively. The resulting precipitates were collected by centrifugation at 8000 rpm, washed three times with ethanol, and finally dried under vacuum at 60 °C for 12 h.

**1.4 Electrochemical measurements**

The electrochemical performances were measured in coin cells. The working electrodes were prepared by mixing polyvinylidene fluoride (PVDF, 10 wt.%), Super P carbon (20 wt.%) and active materials (70 wt.%) in N-methypyrrolidone (NMP) solvent on titanium foil which was used as the current collectors. The coated electrode was dried in vacuum at 60 ℃ for 12 h. The separator was glass fiber. 3 M Zn(CF_3_SO_3_)_2_ aqueous solution as the electrolyte. The cycling performance and rate capability were tested by a battery measurement system (CT3001A, Wuhan Land, China) at room temperature. The cyclic voltammetry (CV) and electrochemical impedance spectroscopy (EIS) were tested on a CHI 760E electrochemical workstation, and the CV curves was obtained in the potential range from 0.3 to 1.5 V.

# **1.5 Computation methods**

The ion diffusion coefficients (D) were estimated through Galvanostatic Intermittent Titration Technique (GITT) and calculated according to the following equation:

$D_{Zn}=\frac{4}{\pi\tau}\left( \frac{n_{m}V_{m}}{S} \right)^{2}\left( \frac{\Delta E_{s}}{\Delta E_{t}} \right)^{2}$ (1)

Here, *τ* is the duration of the current pulse (s); *n_m_* is the number of moles (mol); *V_m_* is the molar volume of the electrode (cm^3^ mol^-1^); *S* is the electrode/electrolyte contact area (cm^2^); Δ*E_s_* is the steady-state voltage change, due to the current pulse and Δ*E_t_* is the voltage change during the constant current pulse, eliminating the iR drop.

The kinetic of capacitive contribution can be obtained through calculating the CV curves at different scan rates. The relationship between current (i) and scan rate (v) can be written as:

$i=av^{b}$ (2)

$\log\left( i \right)=b\cdot\log\left( v \right)+\log\left( a \right)$ (3)

where *i* is the current (A), *v* is the scan rate (mV·s^-1^), and *a* and *b* are adjustable parameters. The value of *b* is from 0.5 to 1, wherein *b*=0.5 indicates a full diffusion controlled process and *b*=1 corresponds to the full capacitive contribution.

$ⅈ\left( V \right)=k_{1}v+k_{2}v^{1/2}$ (4)

where in *k_1_* and *k_2_* are variable parameters. When the voltage is fixed, the response current *i (V)* consists of surface capacitive (*k_1_v*) and diffusion-controlled (*k_2_v^1/2^*) contribution. The following equation can be obtained through equivalent transformation:

$i\left( V \right)/v^{1/2}=k_{1}v^{1/2}+k_{2}$ (5)

There is a linear relationship between *i/v^1/2^* and *v^1/2^*. Thus, *k_1_* and *k_2_* can be concluded by linear fitting. Thereby, the ratio of the capacitive and diffusion-controlled contribution can be obtained.


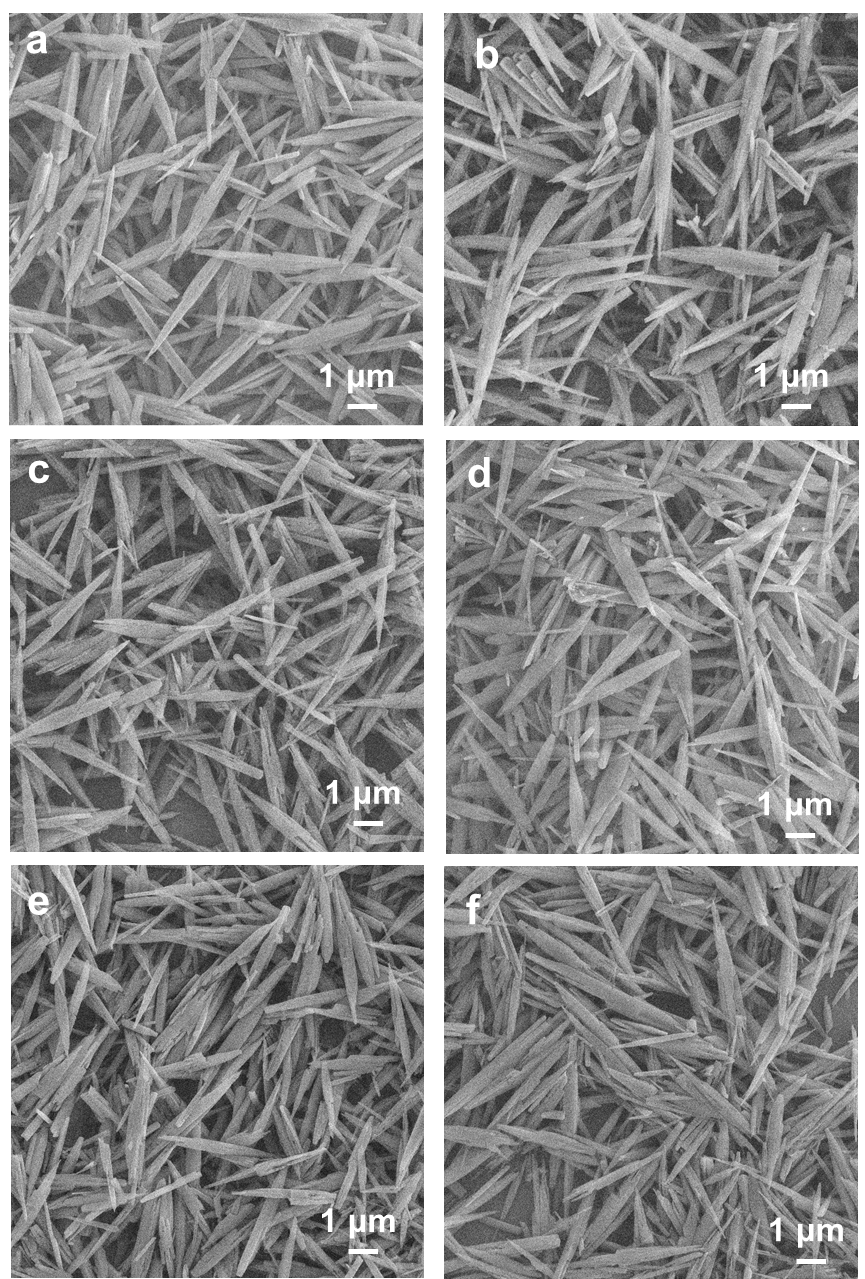


**Figure S1.** SEM images of (a) Br@P-0.5, (b) Br@P-1, (c) Br@P-2, (d) Br@P-4, (e) Br@P-8, and (f) Br@P-24.


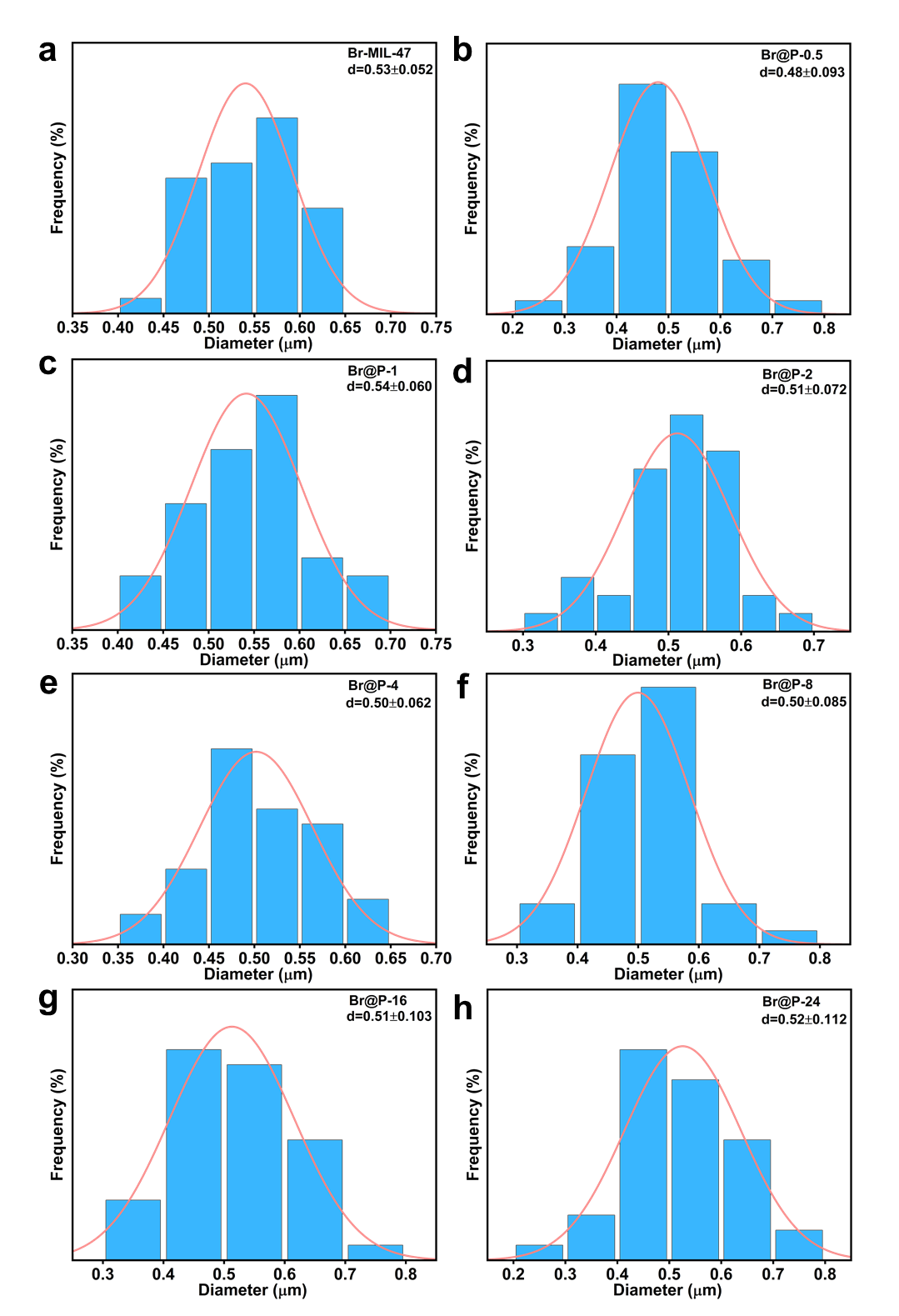


**Figure S2.** Particle size distribution diagrams of (a) Br-MIL-47, (b) Br@P-0.5, (c) Br@P-1, (d) Br@P-2, (e) Br@P-4, (f) Br@P-8, (g) Br@P-16, and (h) Br@P-24.


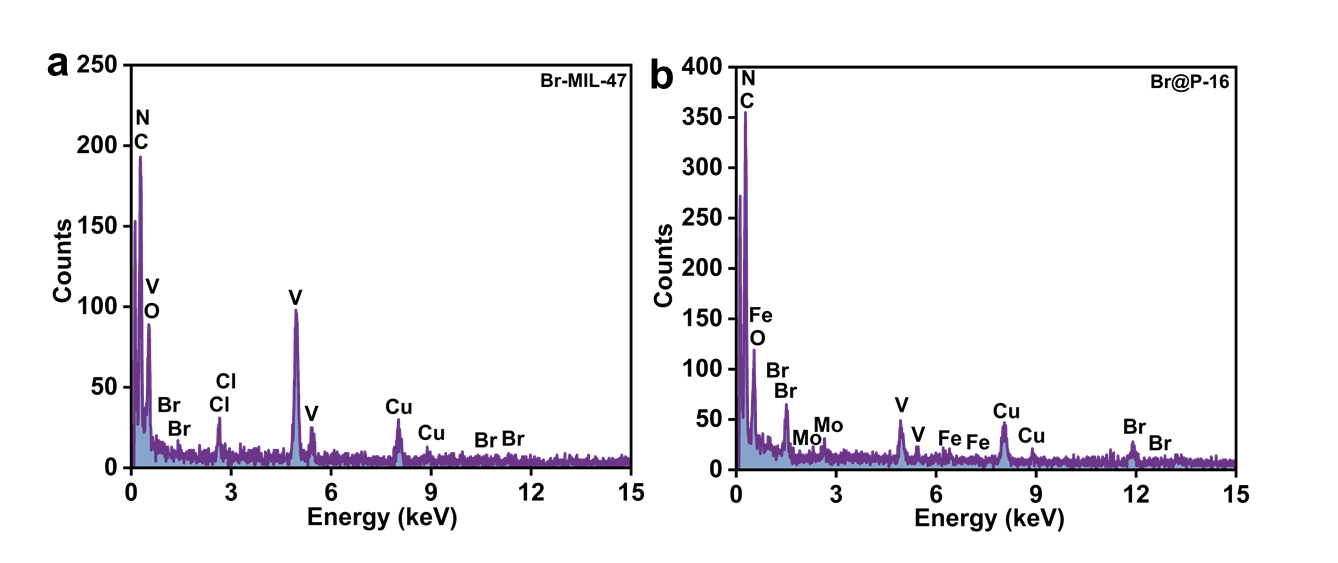


**Figure S3.** EDX images of Br-MIL-47 and Br@P-16.


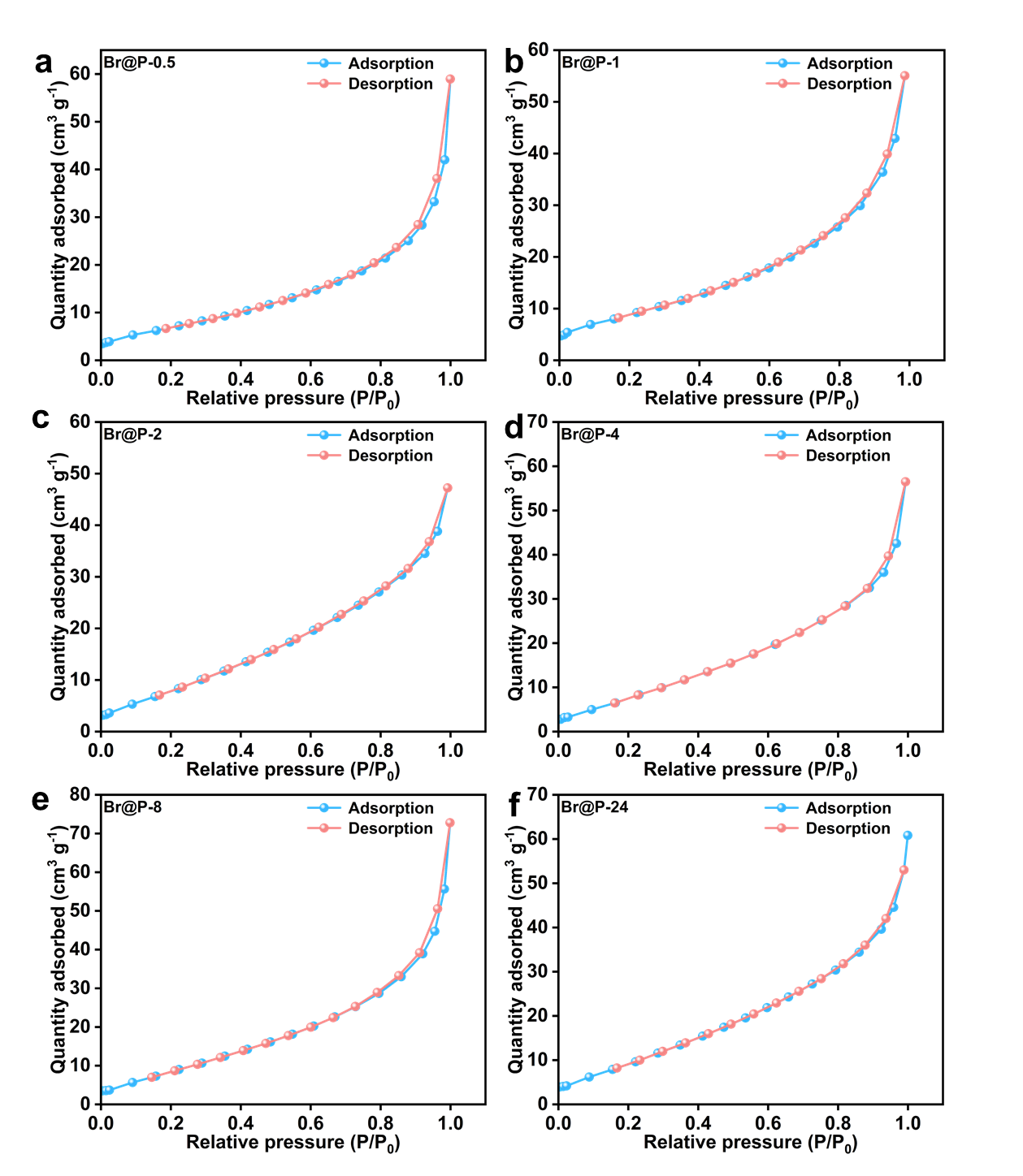


**Figure S4.** N_2_ adsorption–desorption isotherms of (a) Br@P-0.5, (b) Br@P-1, (c) Br@P-2, (d) Br@P-4, (e) Br@P-8, and (f) Br@P-24.


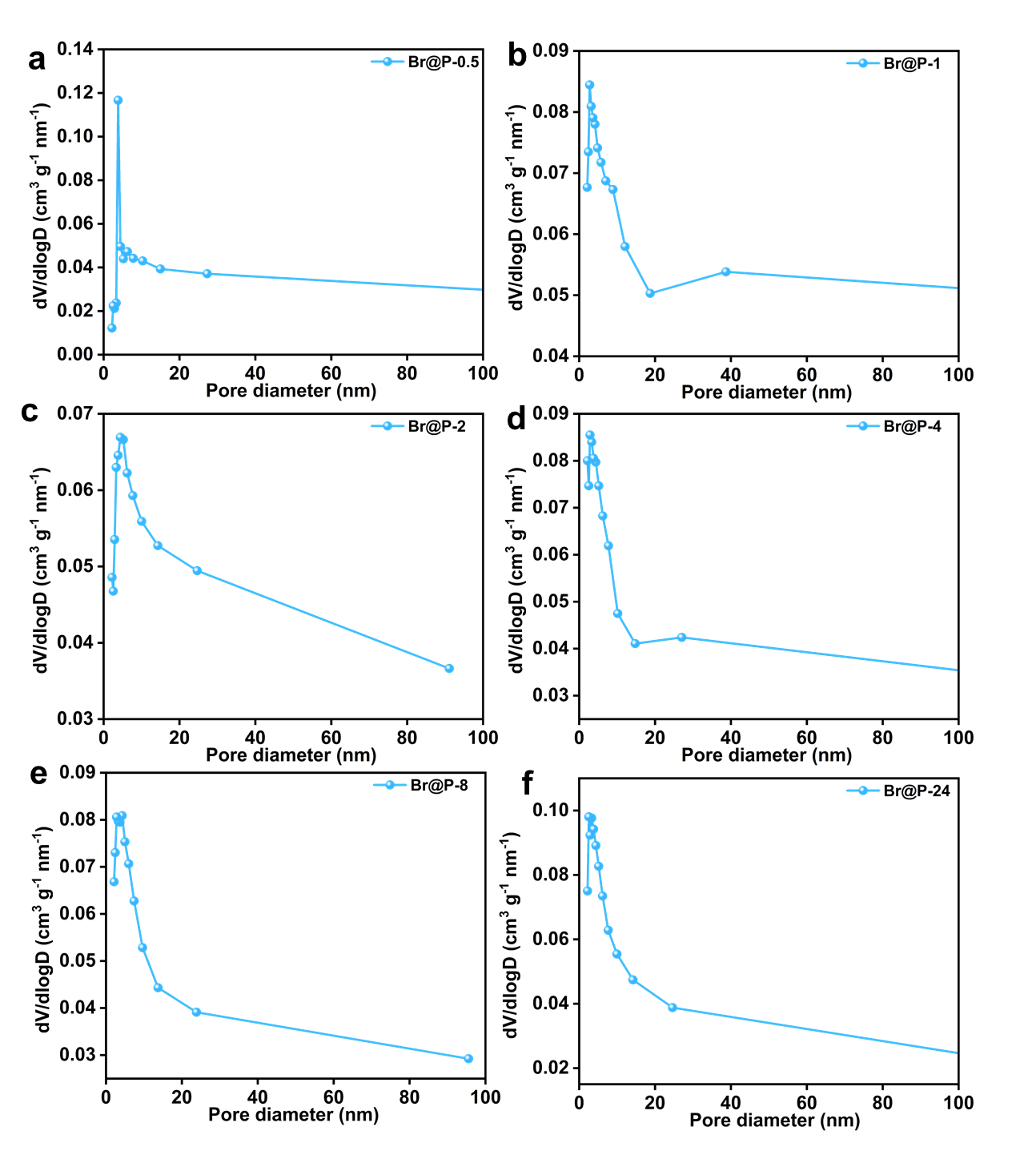


**Figure S5.** Pore size distribution plots of (a) Br@P-0.5, (b) Br@P-1, (c) Br@P-2, (d) Br@P-4, (e) Br@P-8, and (f) Br@P-24.


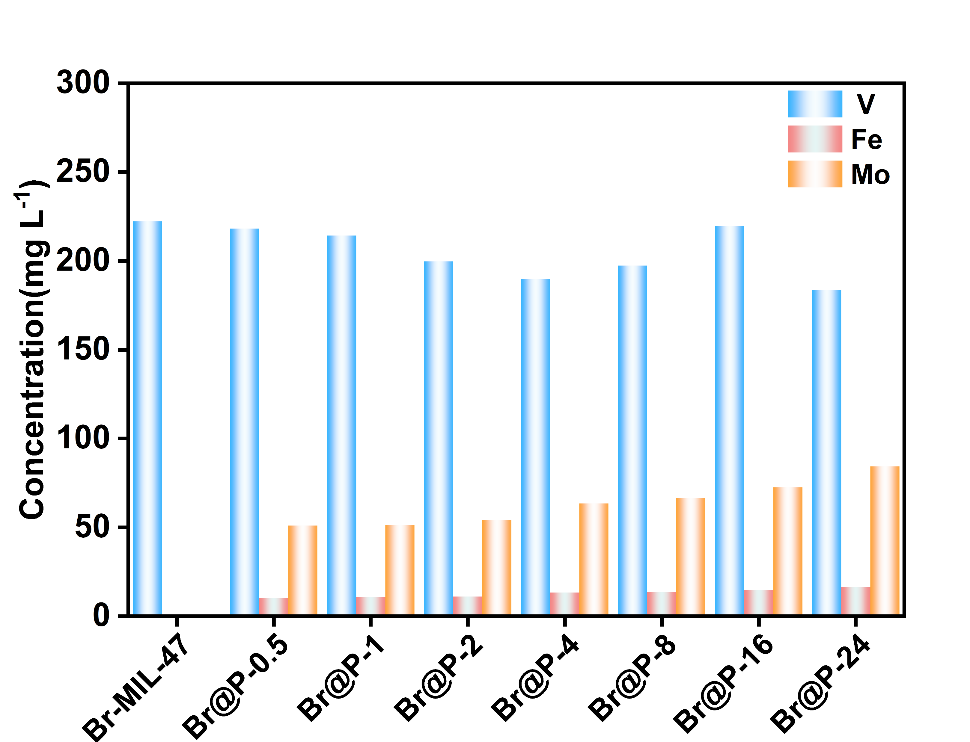


**Figure S6.** Metal ions concentration of Br-MIL-47 and Br@P-X.


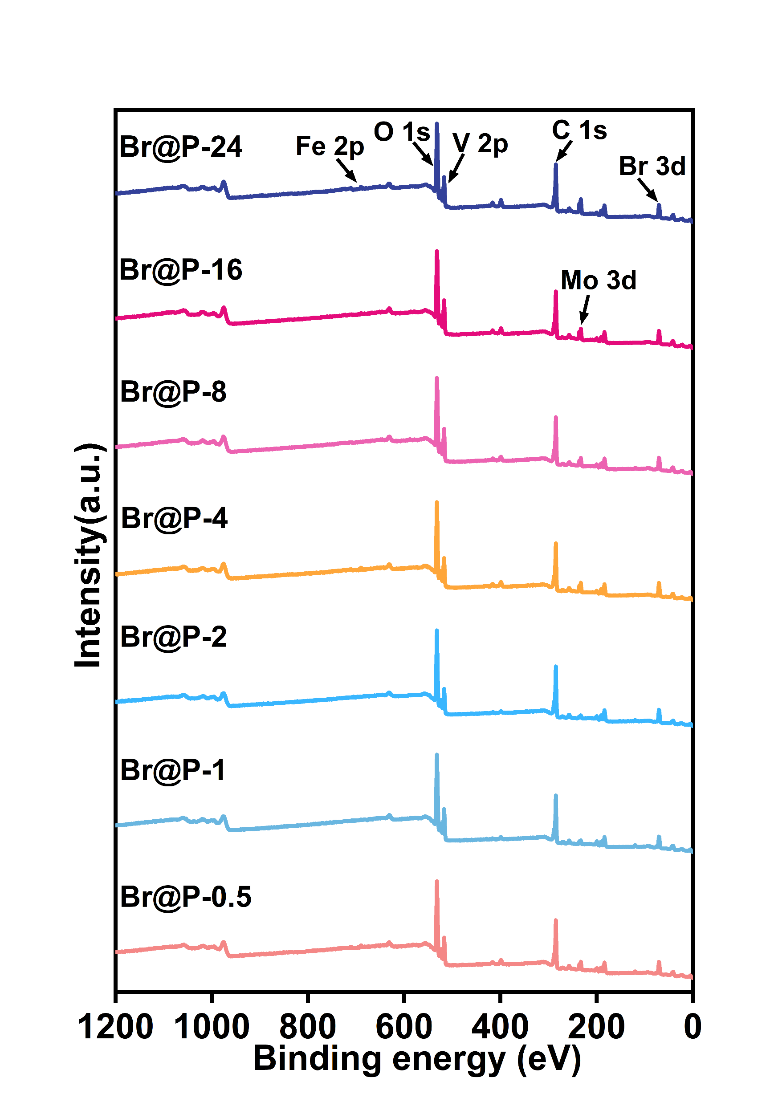


**Figure S7.** (a) Full XPS spectra of Br@P-X.


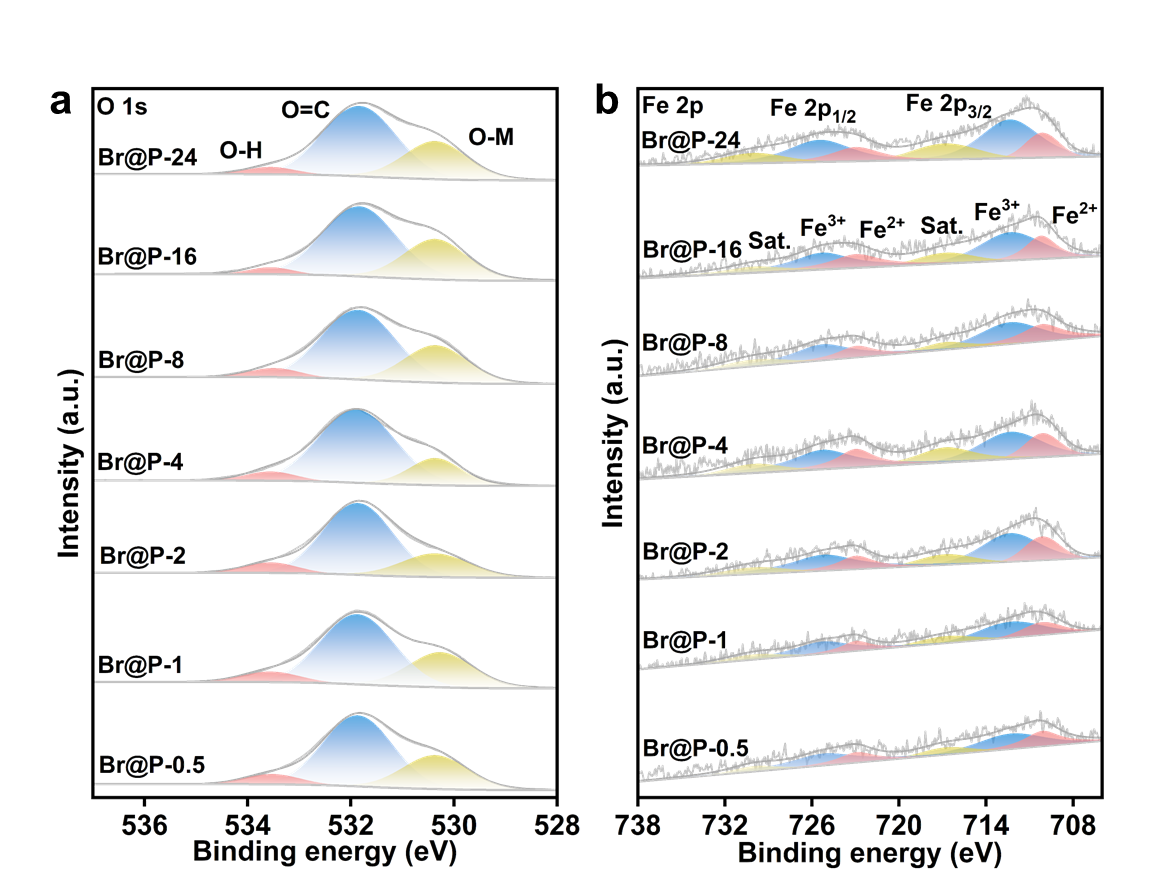


**Figure S8.** (a) O 1s XPS spectra of Br@P-X. (b) Fe 2p XPS spectra of Br@P-X.


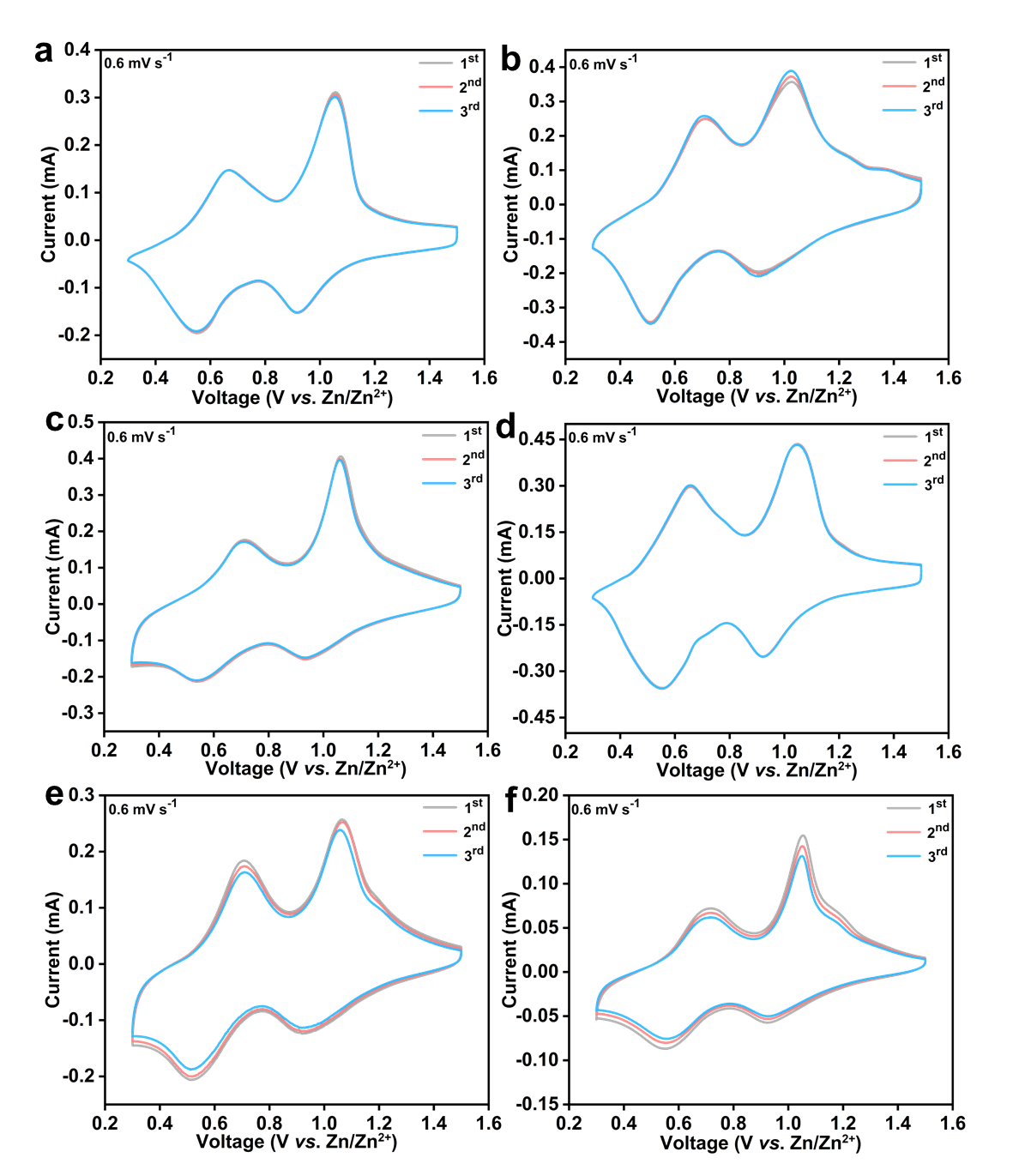


**Figure S9.** CV curves of (a) Br@P-0.5, (b) Br@P-1, (c) Br@P-2, (d) Br@P-4, (e) Br@P-8, and (f) Br@P-24 cathodes at 0.6 mV s^–1^, respectively.


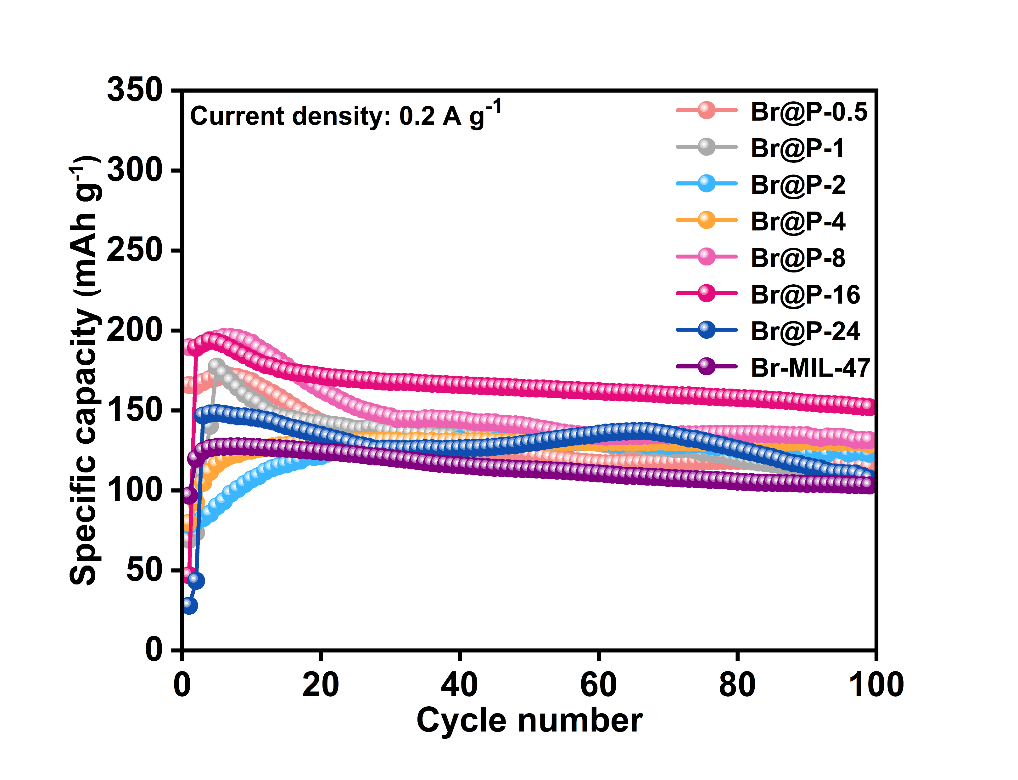


**Figure S10.** Comparison of cycling performance of Br@P-X cathodes at a current density of 0.2 A g^-1^.


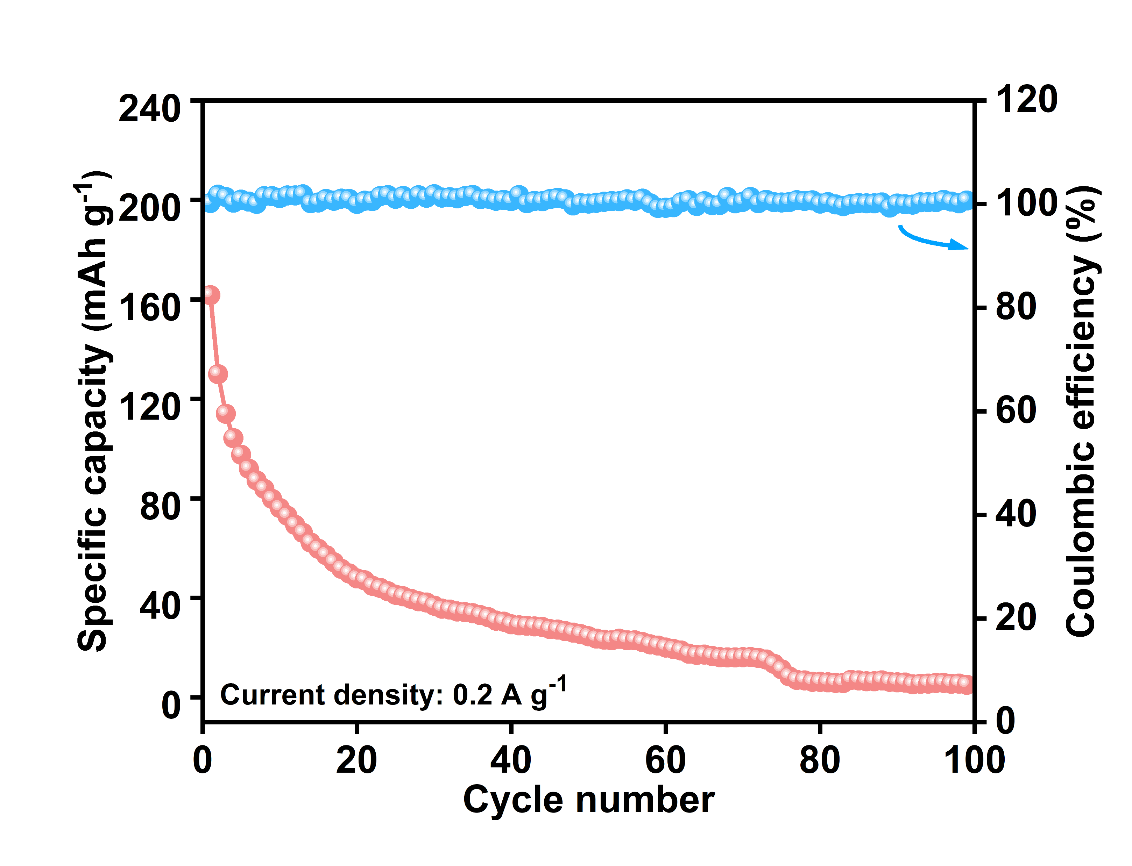


**Figure S11.** Cycling performance of FeMo_6_ cathode at a current density of 0.2 A g^-1^.


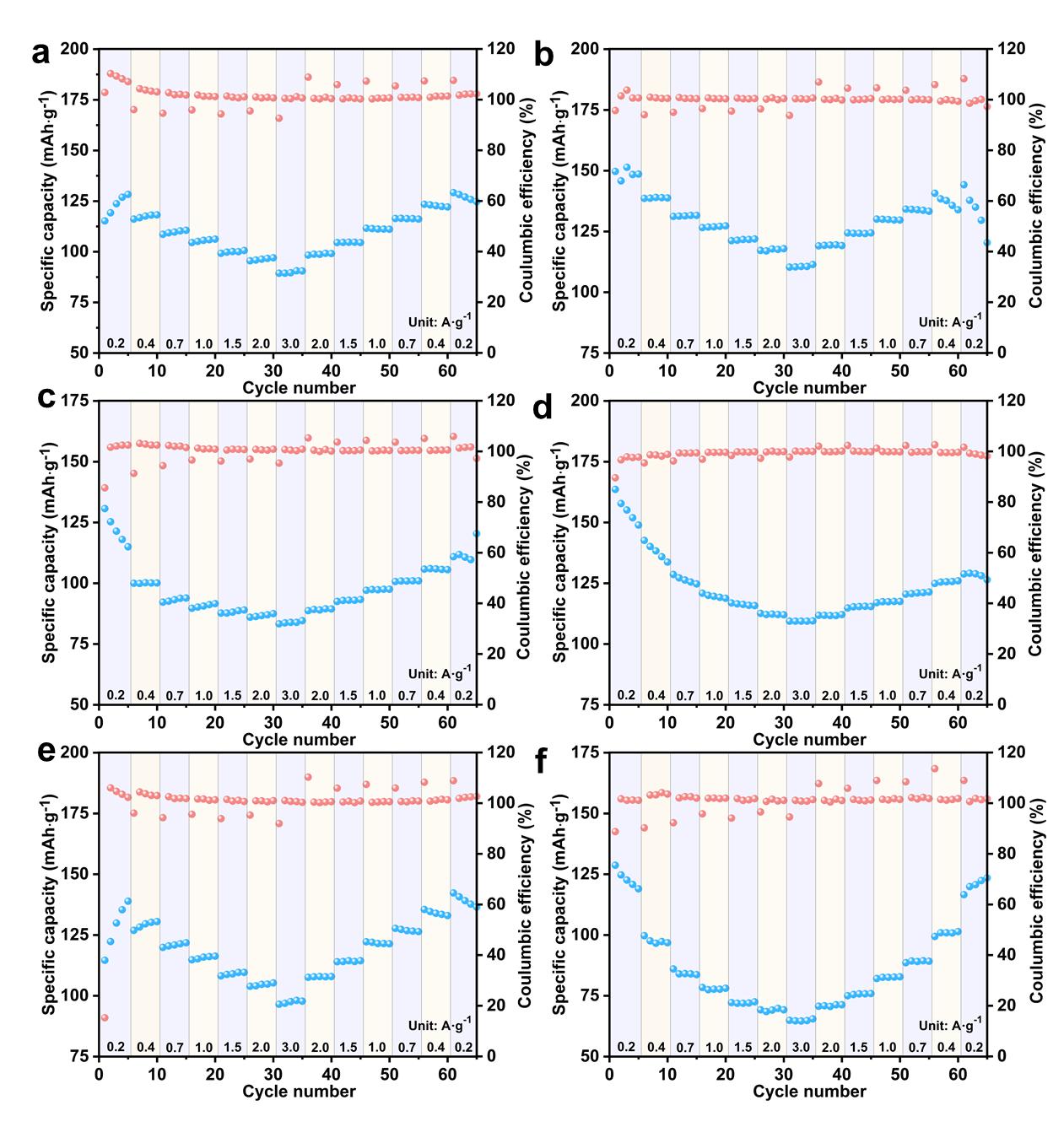


**Figure S12.** Rate capabilities of (a) Br@P-0.5, (b) Br@P-1, (c) Br@P-2, (d) Br@P-4, (e) Br@P-8, and (f) Br@P-24 cathodes.


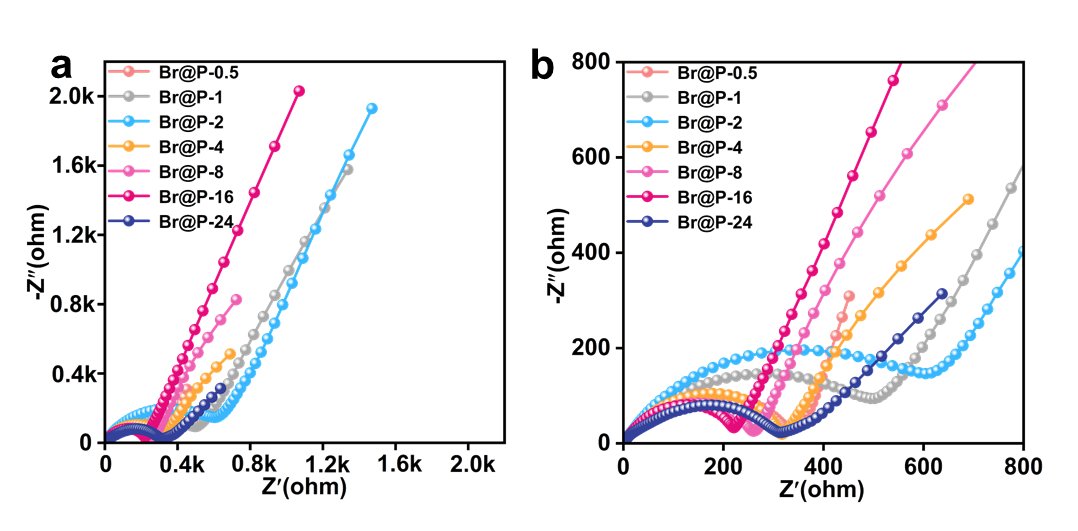


**Figure S13.** (a) EIS spectra of Br@P-X cathodes. (b) The enlarged EIS spectra from (a).


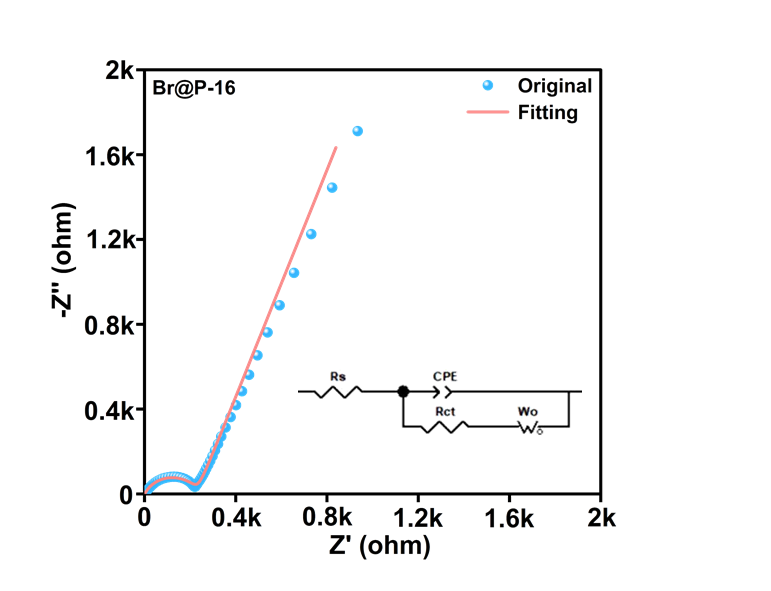


**Figure S14.** EIS fitting spectra of Br@P-16.

*
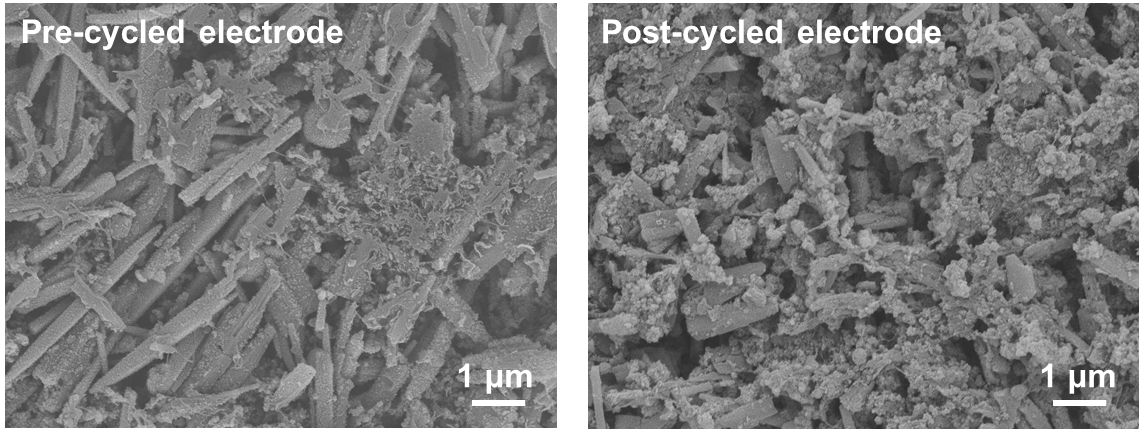
*

**Figure S15**. Br@P-16 electrode before and after cycling.


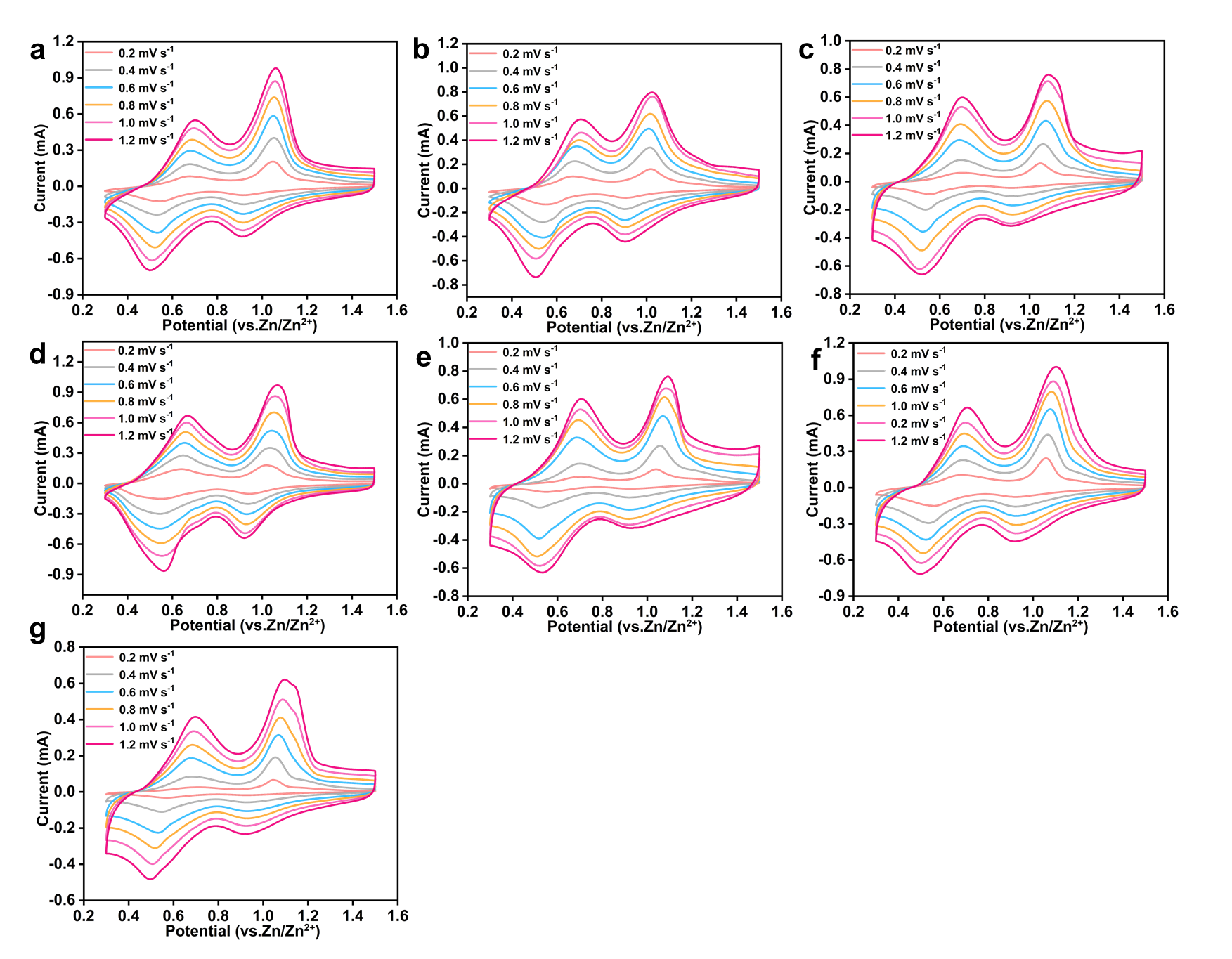


**Figure S16.** The CV curves at different scan rates of of (a) Br@P-0.5, (b) Br@P-1, (c) Br@P-2, (d) Br@P-4, (e) Br@P-8, (f) Br@P-16, and (g) Br@P-24 cathodes.


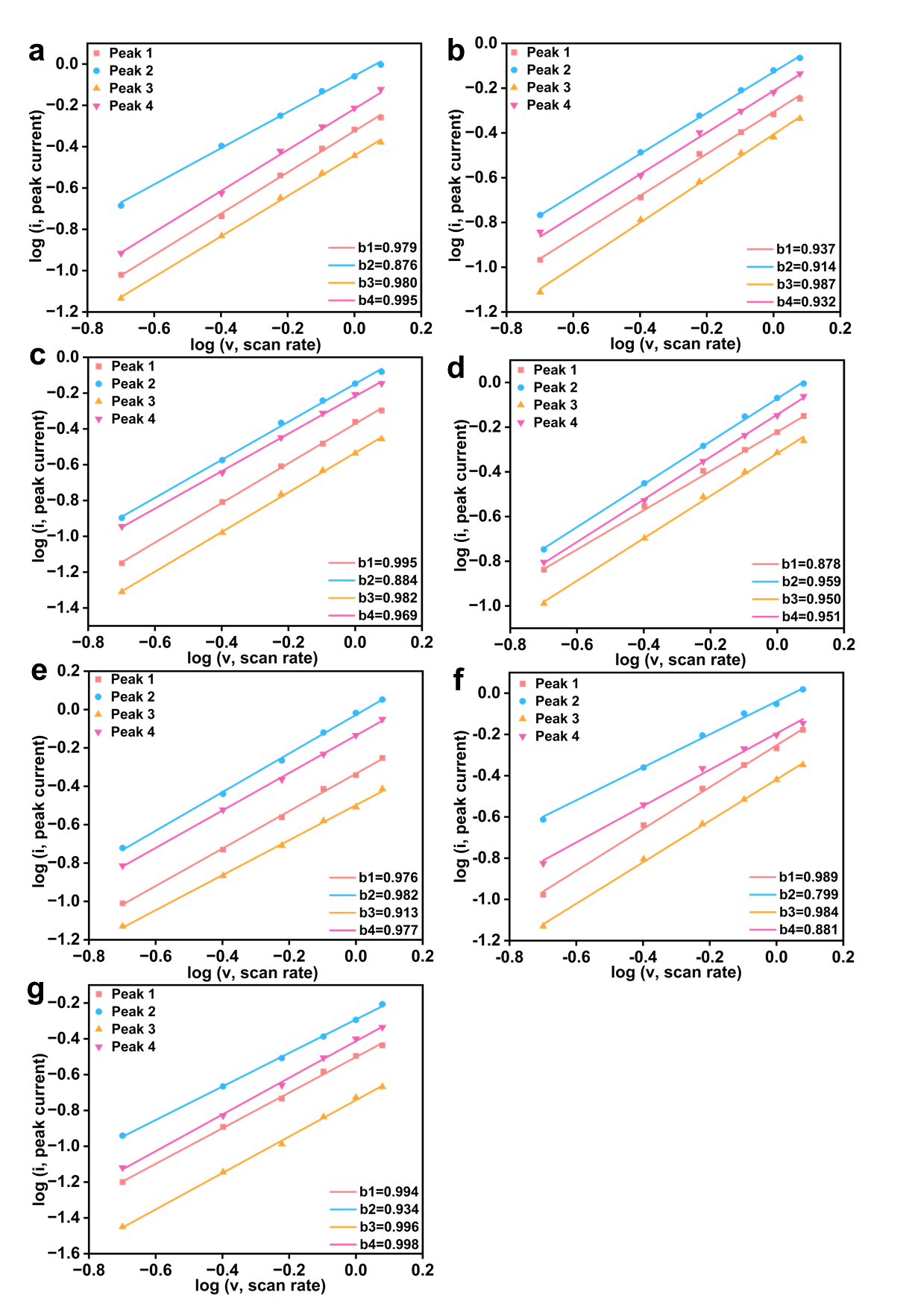


**Figure S17.** Corresponding *Log i* versus *log ν* plots of the cathodic and anodic current response at the two pairs of peaks shown in CV curves of the (a) Br@P-0.5, (b) Br@P-1, (c) Br@P-2, (d) Br@P-4, (e) Br@P-8, (f) Br@P-16, and (g) Br@P-24 cathodes.


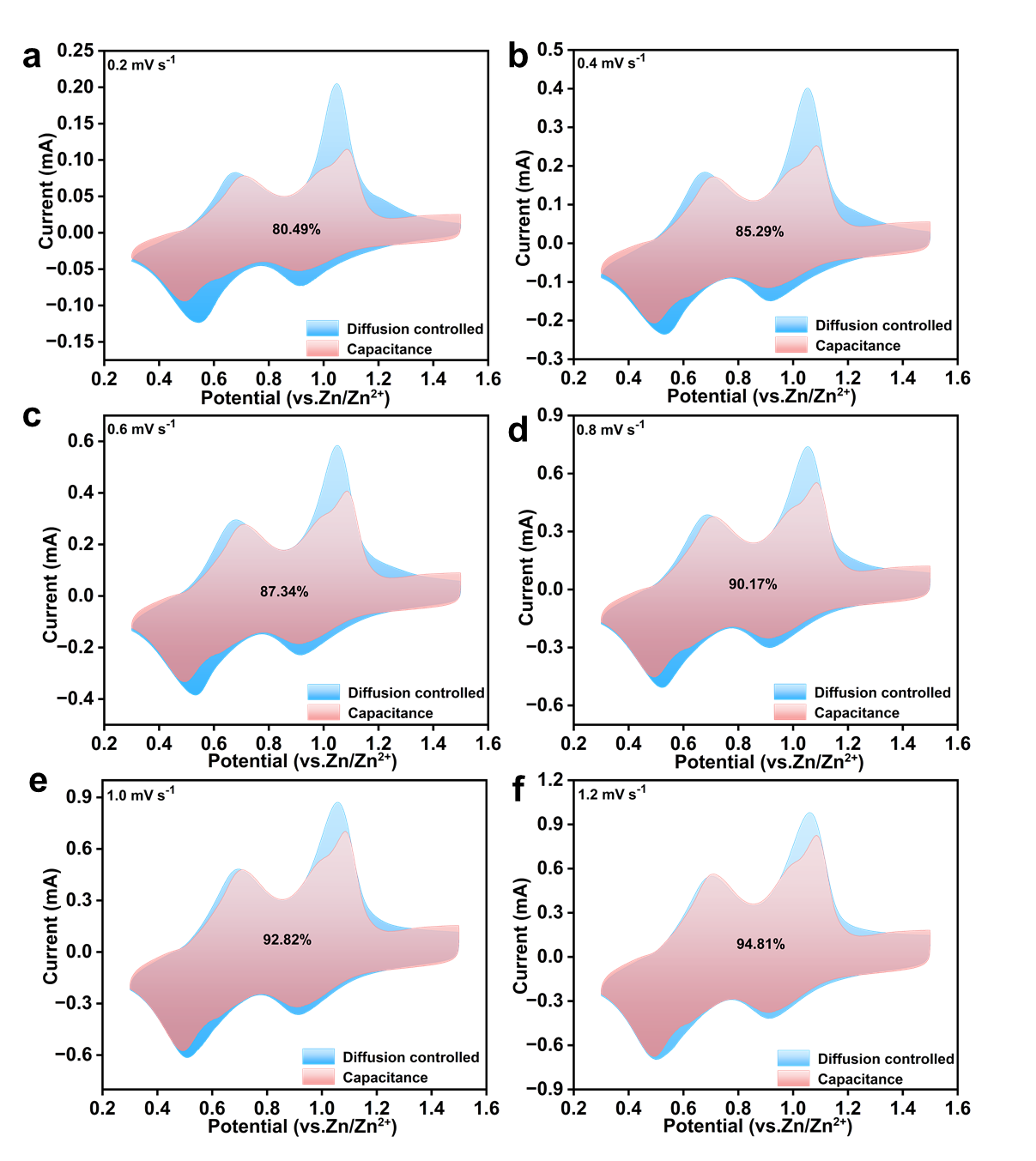


**Figure S18.** The capacitive contributions of the Br@P-0.5 electrode at different scan rates.


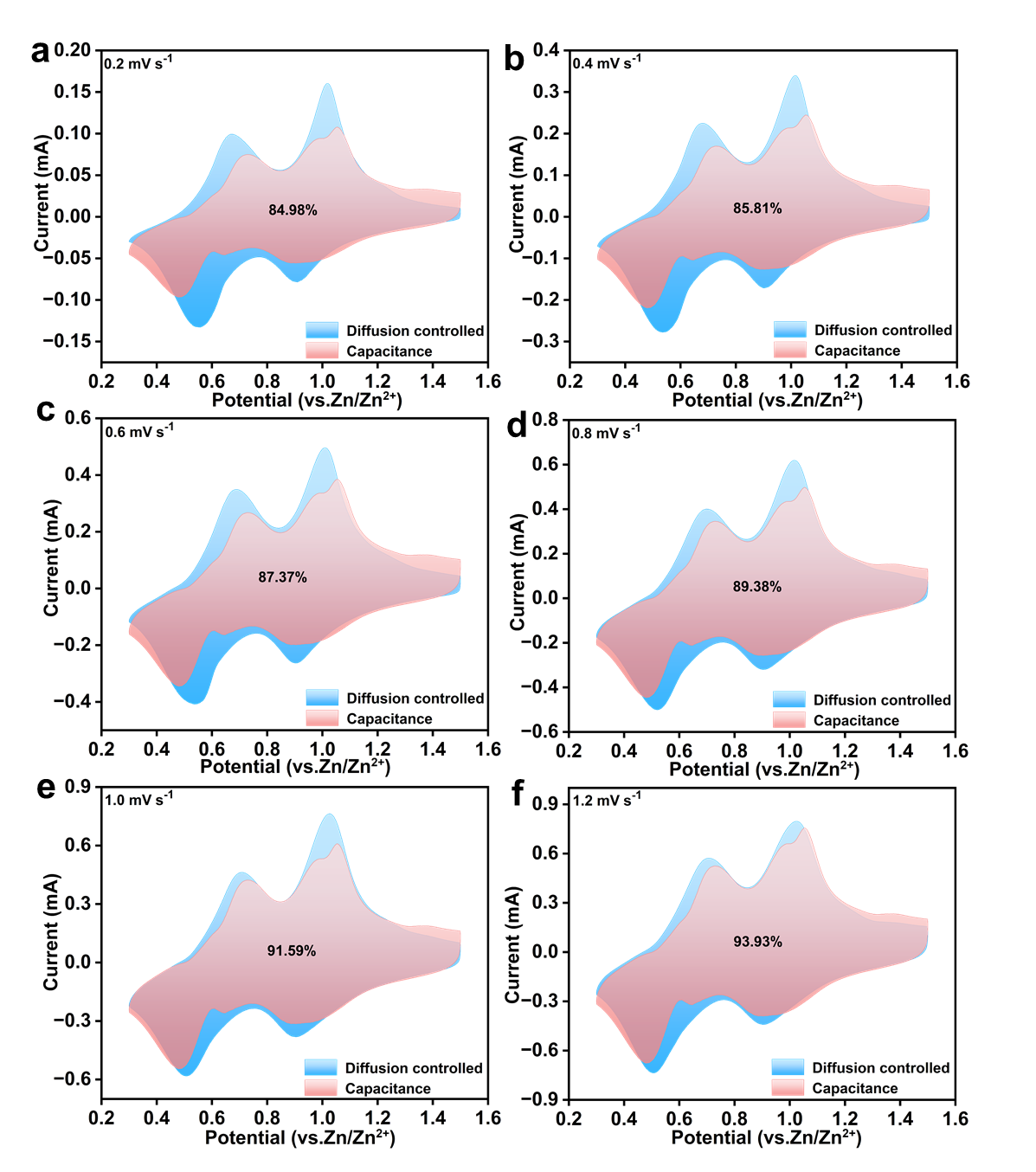


**Figure S19.** The capacitive contributions of the Br@P-1 electrode at different scan rates.


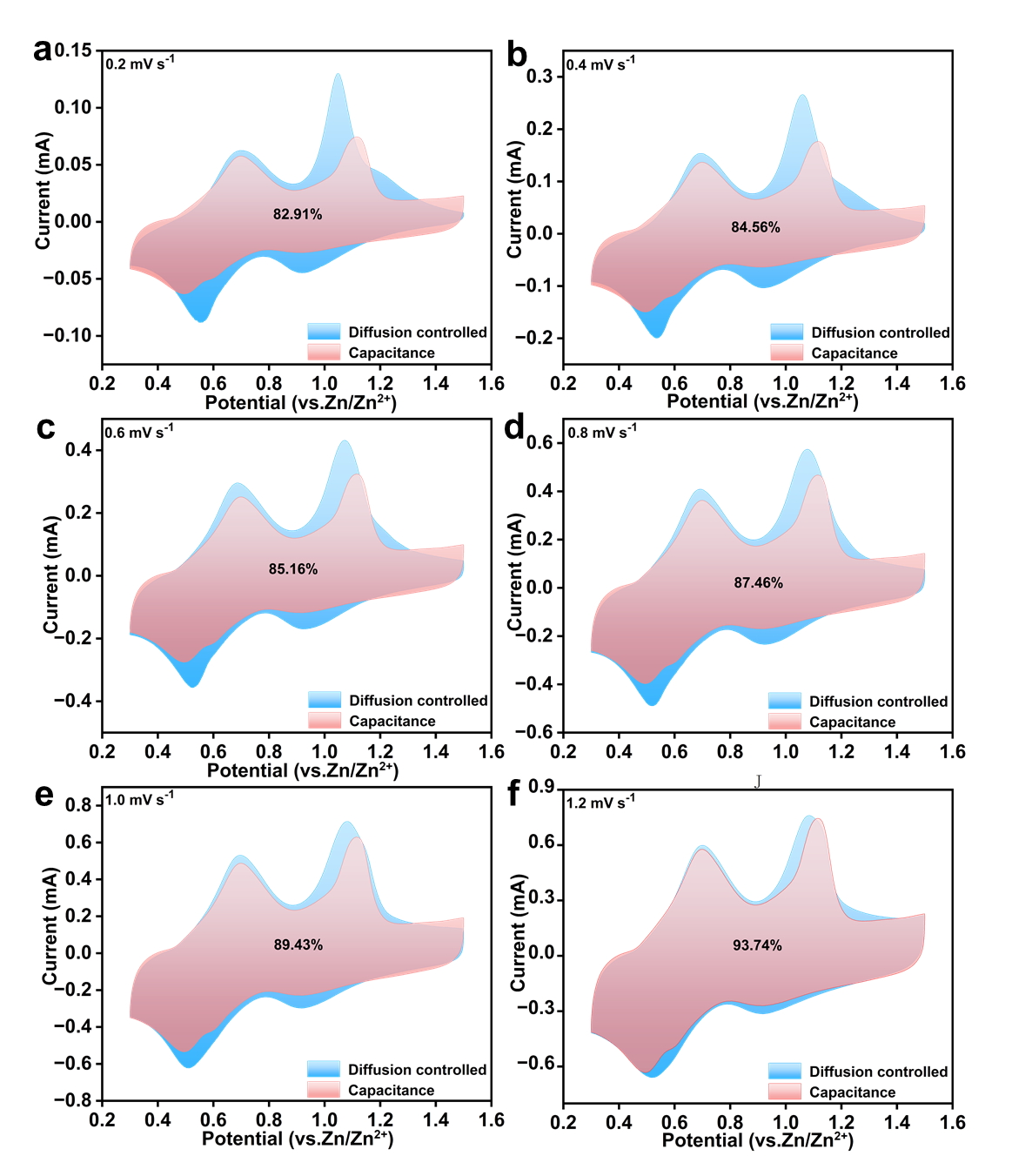


**Figure S20.** The capacitive contributions of the Br@P-2 electrode at different scan rates.


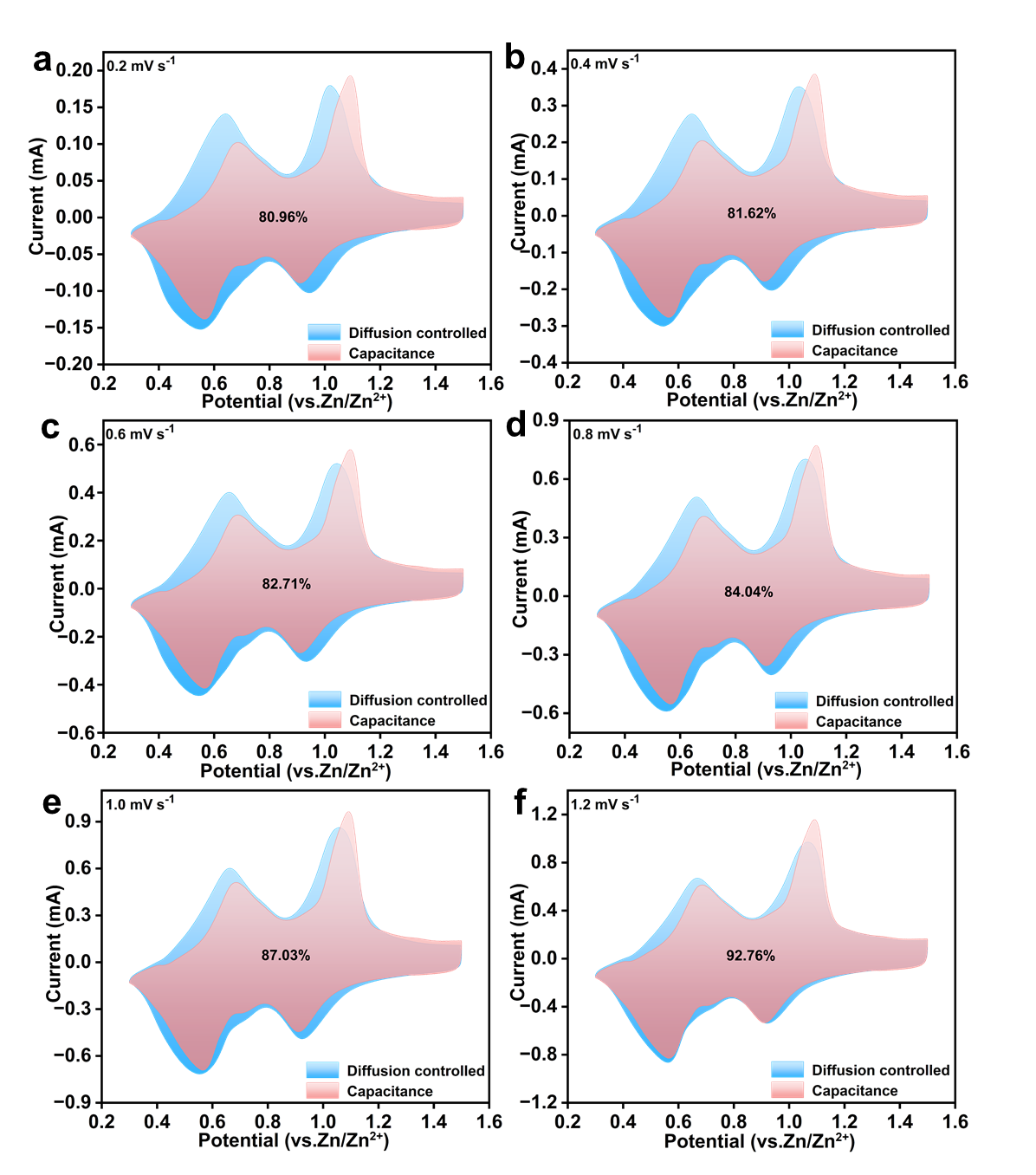


**Figure S21.** The capacitive contributions of the Br@P-4 electrode at different scan rates.


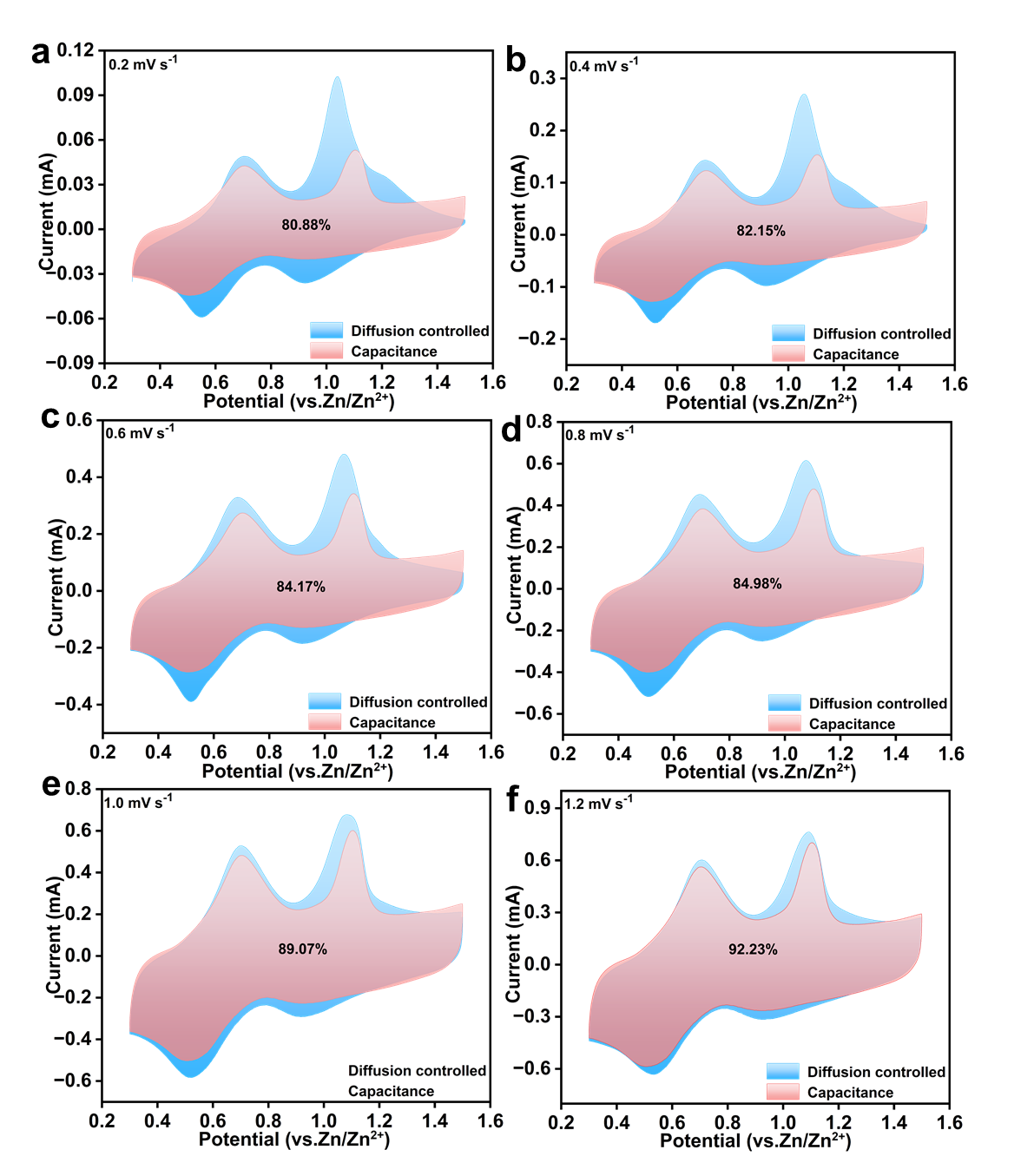


**Figure S22.** The capacitive contributions of the Br@P-8 electrode at different scan rates.


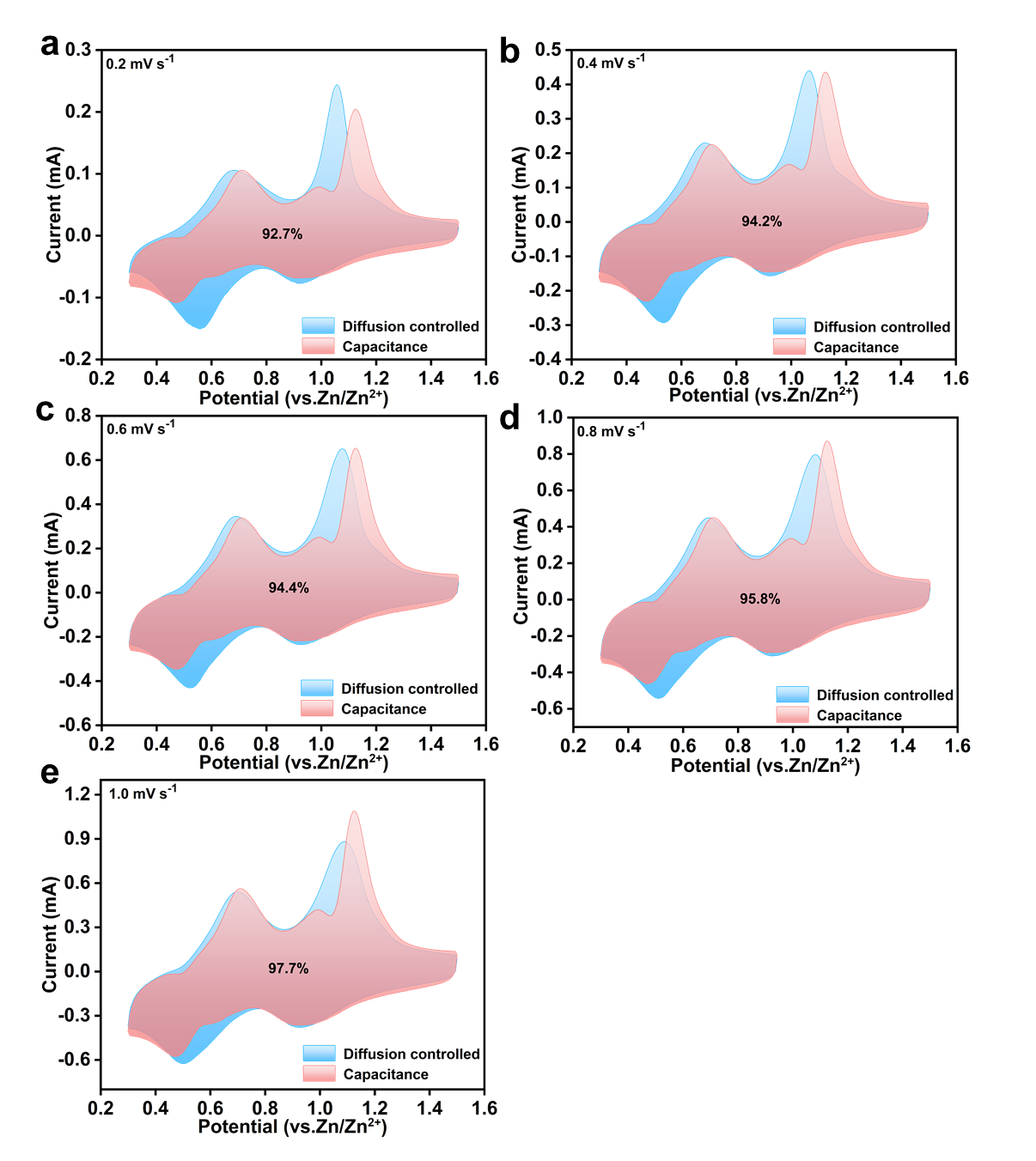


**Figure S23.** The capacitive contributions of the Br@P-16 electrode at different scan rates.


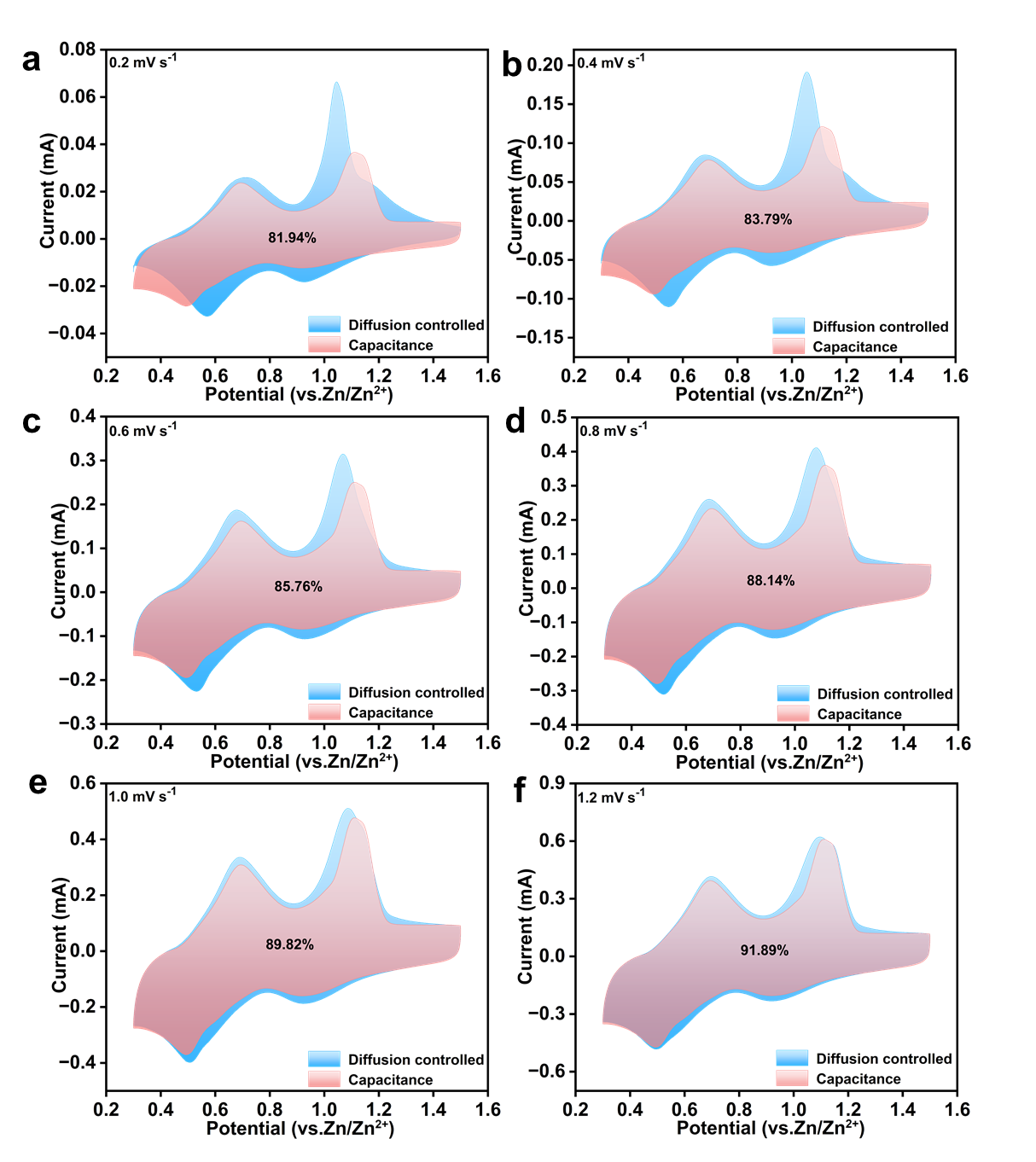


**Figure S24.** The capacitive contributions of the Br@P-24 electrode at different scan rates.


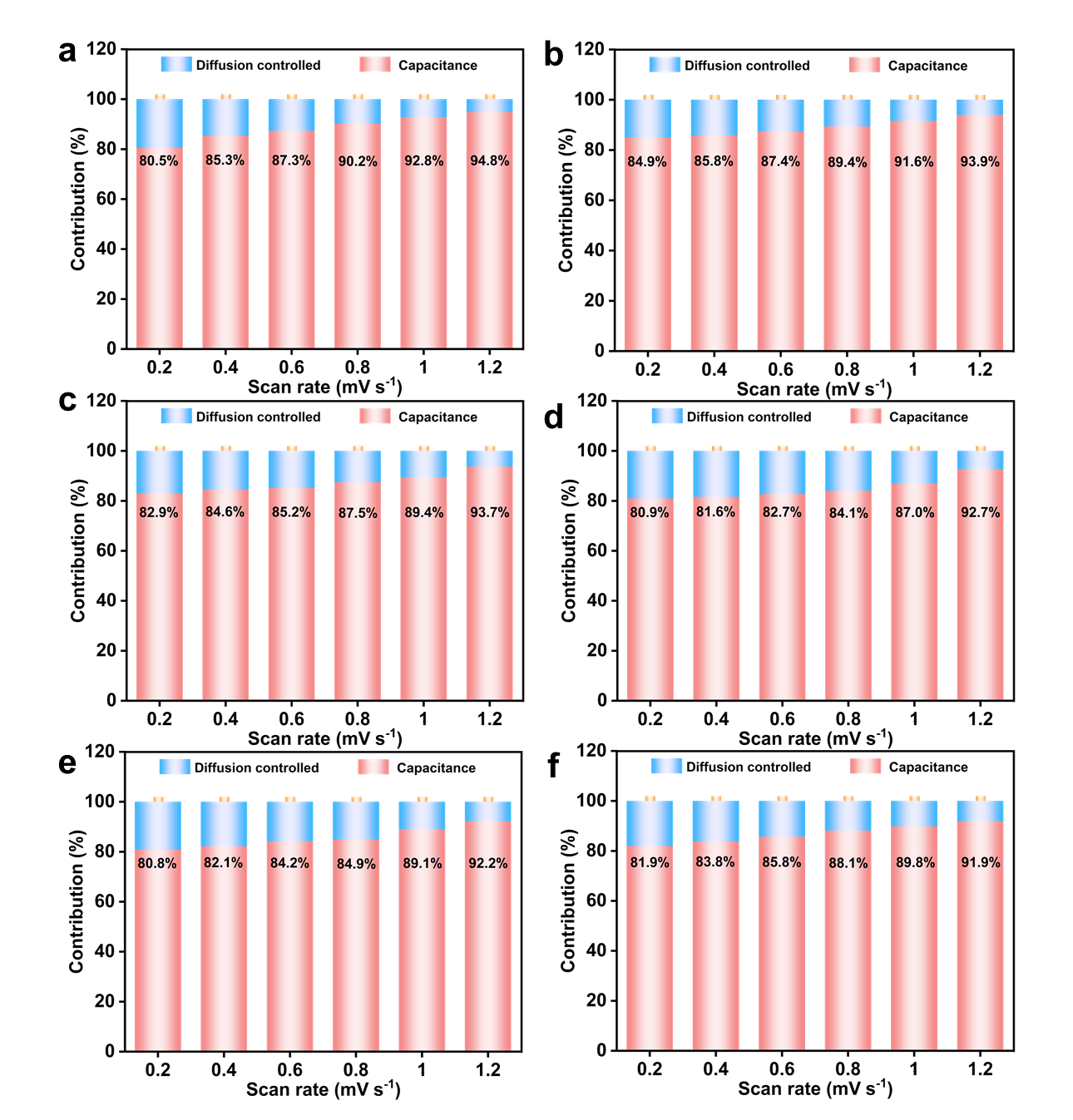


**Figure S25.** The capacitive contributions of the (a) Br@P-0.5, (b) Br@P-1, (c) Br@P-2, (d) Br@P-4, (e) Br@P-8, and (f) Br@P-24 cathodes calculated at different scan rates.


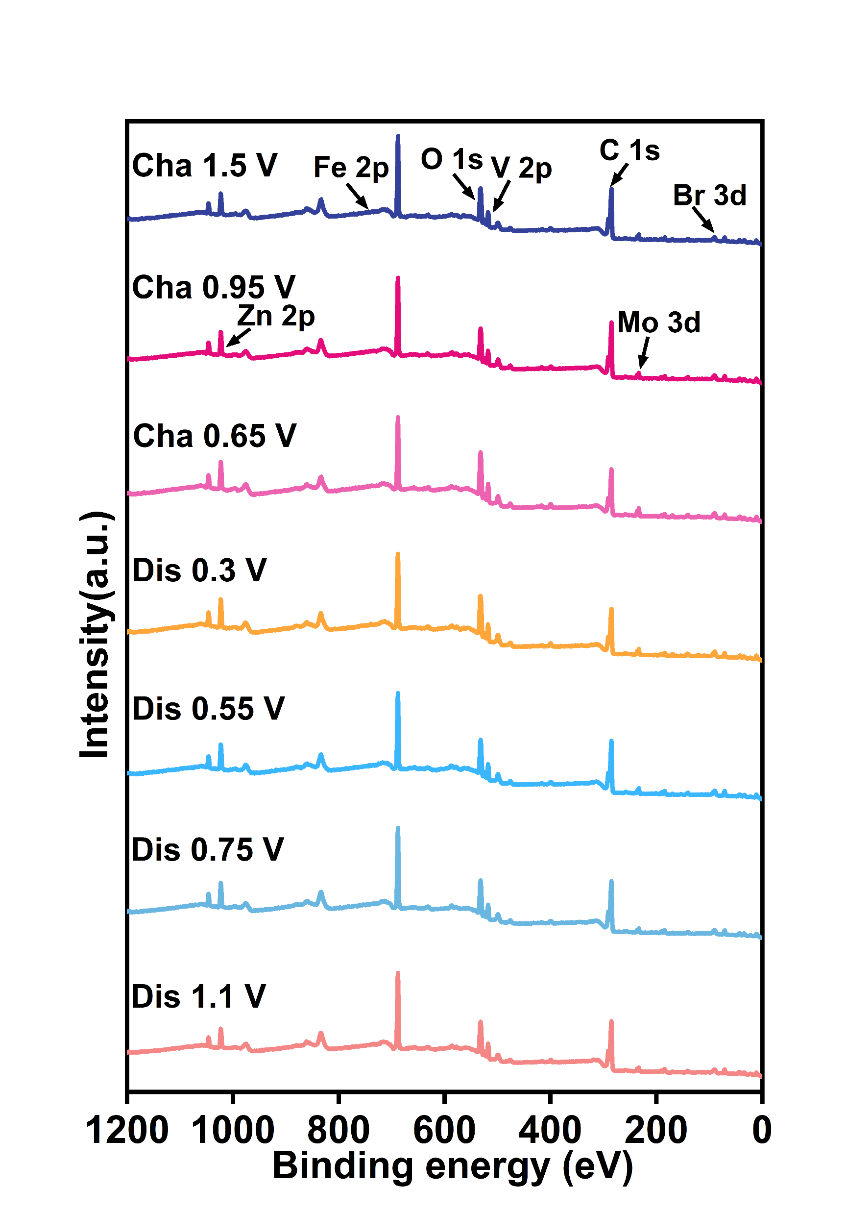


**Figure S26.** Full XPS spectra of Br@P-16 cathode during charging and discharging process.


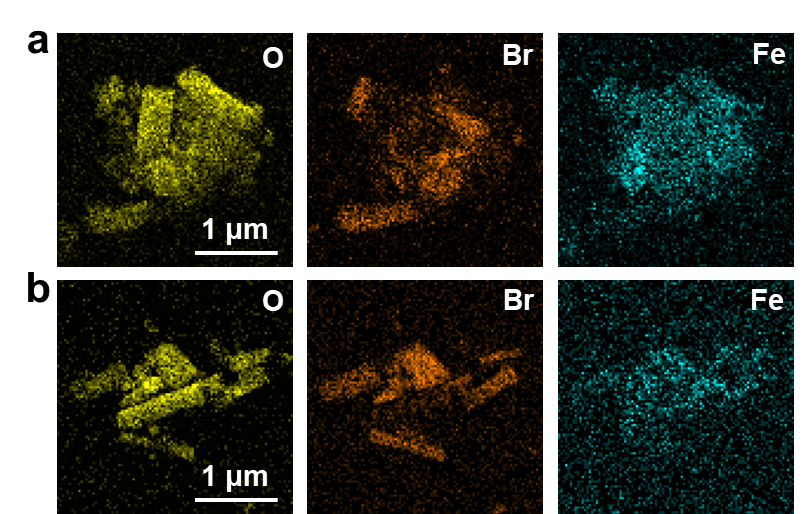


**Figure S27.** Elemental mapping images of the Br@P-16 cathode at (a) discharge 0.3 V and (b) charge 1.5 V.


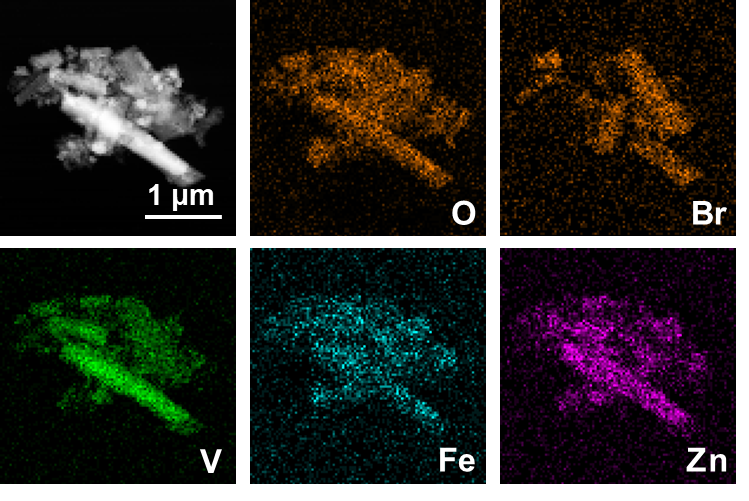


**Figure S28.** Elemental mapping images of the Br@P-16 cathode at discharge 0.75V.


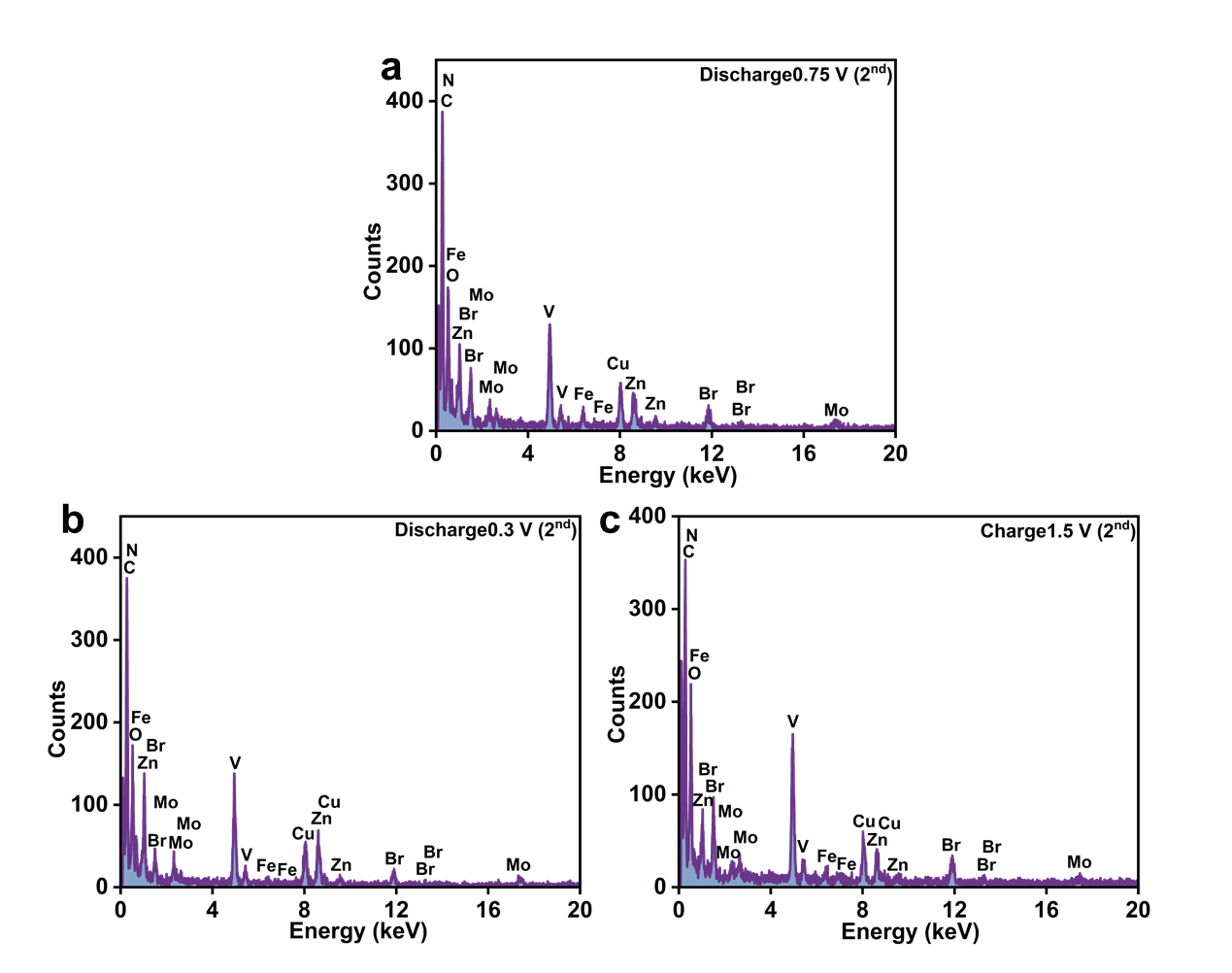


**Figure S29.** EDX of the Br@P-16 cathode at (a) discharge 0.75 V, (b) discharge 0.3 V and (c) charge 1.5 V.

**
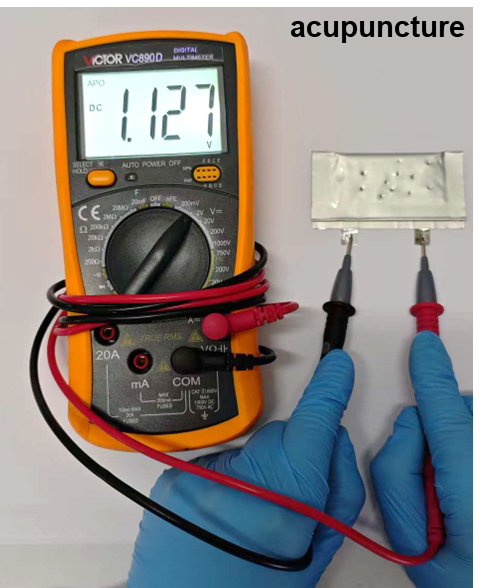
**

**Figure S30.** The open circuit voltage of Br@P-16 soft pack battery under acupuncture states.


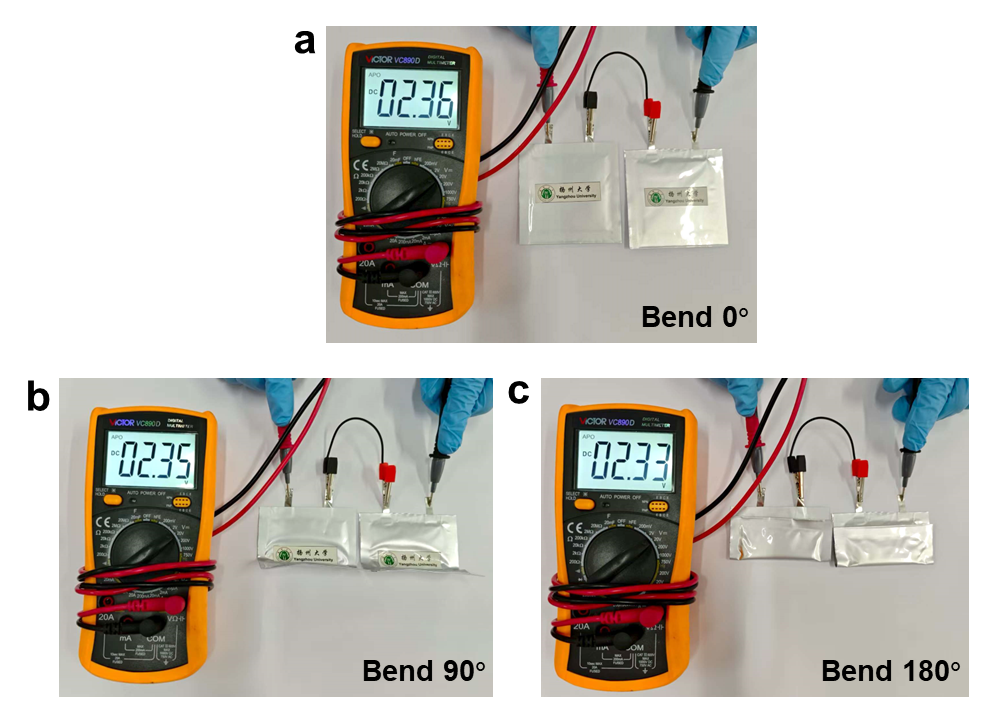


**Figure S31.** Open-circuit voltage of two series-connected Zn/Br@P-16 soft-pack batteries under (a) 0°, (b) 90°, and (c) 180° bending states.


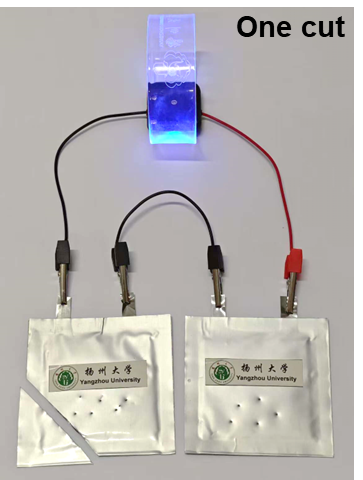


**Figure S32.** Br@P-16 soft pack battery supply power to wristband.

**Table S1** The physical properties of as-prepared samples.

| **Samples** | **S_BET_ (m^2^ g^-1^)** | **Average pore size (nm)** |
| --- | --- | --- |
| Br-MIL-47 | 24.06 | 11.00 |
| Br@P-0.5 | 27.50 | 10.71 |
| Br@P-1 | 34.34 | 10.08 |
| Br@P-2 | 36.84 | 9.91 |
| Br@P-4 | 37.67 | 9.00 |
| Br@P-8 | 38.98 | 8.17 |
| Br@P-16 | 39.56 | 7.84 |
| Br@P-24 | 42.24 | 7.76 |

**Table S2.** Comparison of the Br@P-16 cathode with reported aqueous zinc-ion batteries.

| **Samples** | **Current density**  **/A g^-1^** | | **Cycles** | **Discharge Capacity**  **/mAh g^-1^** | **References** |
| --- | --- | --- | --- | --- | --- |
| Mn-MOF-74 | | 0.5 | 500 | 86.5 | [2] |
| V-MOF | | 1 | 70 | 86 | [3] |
| MIL-88B(V)@rGo | | 2 | 400 | 290 | [4] |
| Cu-TBPQ | | 2 | 500 | 136.7 | [5] |
| VBr-180 | | 2 | 3000 | 74.9 | [6] |
| Mn-TOC | | 0.05 | 100 | 78.7 | [7] |
| MnBTC69 | | 1 | 1000 | 25.7 | [8] |
| Cu-HHTP/MX | | 4 | 1000 | 166.9 | [9] |
| Cu_3_(HHTP)_2_ | | 4 | 500 | 93.3 | [10] |
| MOF-73 | | 0.3 | 1000 | 137 | [11] |
| Mn-1,4-DHAQ | | 1 | 1000 | 60.2 | [12] |
| Mn-H_3_BTC-MOF-4 | | 0.1 | 100 | 138 | [13] |
| Mn(BTC) | | 1 | 900 | 42.32 | [14] |
| **Br@P-16** | | **3** | **2500** | **95.6** | **This work** |

**References**

[1] B. Ravel, M. Newville, *J. Synchrotron Radiat.* **2005**, 12, 537.

[2] S. Deng, B. Xu, J. Zhao, C. Kan, X. Liu, *Angew. Chem. Int. Ed*. **2024**, e202401996.

[3] Y. Ru, S. Zheng, H. Xue, H. Pang, *Mater. Today Chem*., **2021**, *21*, 100513.

[4] D. Jia, Z. Shen, Y. Lv, Z. Chen, H. Li, Y. Yu, J. Qiu, X. He, *Adv. Funct. Mater.* **2023**, *34*, 2308319.

[5] J. Liu, Y. Zhou, G. Xing, M. Qi, Z. Tang, O. Terasaki, L. Chen, *Adv. Funct. Mater.* **2024***, 34,* 2312636*.*

[6] Y. Zhang, Q. Li, W. Feng, S. Gao, H. Yue, Y. Su, H. Zhou, J. Huang, L. Han, M. Shakouri, Y. Wang, H. Pang, *Adv. Mater.*, **2025***,* 2507609.

[7] Z. Chang, M. Zhu, Z. Li, S. Wu, S. Yin, Y. Sun, W. Xu, *Small*, **2024***, 20,* 2400923.

[8] Q. Li, Y. Zhang, X. Guo, Z. Yang, Y. Wang, Y. Chen, Y. Liu, H. Yue, S. Gao, H. Zhou, J. Huang, M. Shakouri, Y. Wang, G. Zhu, Z. Liu, Y. Zhang, H. Pang, *Angew. Chem. Int. Ed.* **2025***,* e202509741.

[9] Y. Wang, J. Song, W. Wong, A*ngew. Chem. Int. Ed.* **2023***, 62,* e202218343*.*

[10] K.W. Nam, S.S. Park, R. Reis, V.P. Dravid, H. Kim, C.A. Mirkin, J. F. Stoddart, *Nat. Commun.*, **2019**, *10*, 4948.

[11] W. Gou, H. Chen, Z. Xu, Y. Sun, X. Han, M. Liu, Y. Zhang, *Energy Adv.,* **2022***, 1,* 1065–1070.

[12] Q. Li, Y. Zhang, W. Feng, J. Huang, S. Wei, G. Chen, Y. Liu, M. Du, C. Yin, Z. Yang, Y. Sun, S. Cao, C. Pei, H. Chen, H. Pang, *Adv. Mater.* **2025***,* 2507951.

[13] C. Yin, C. Pan, X. Liao, Y. Pan, L. Yuan, *ACS Appl. Mater. Interfaces,* **2021***, 13,* 35837-35847.

[14] X. Pu, B. Jiang, X. Wang, W. Liu, L. Dong, F. Kang, C. Xu, *Nano-Micro Lett.* **2020***, 12,*152.
