# Supplementary figures and images for: Host‐Guest Synergistic Regulation in Functionalized Metal‐Organic Frameworks for Efficient Aqueous Zinc‐Ion Batteries
Source: Adv Sci (Weinh). 2025 Aug 11;12(42):e11198. doi: 10.1002/advs.202511198 (PMC12622505; doi:10.1002/advs.202511198)

## Slide 1
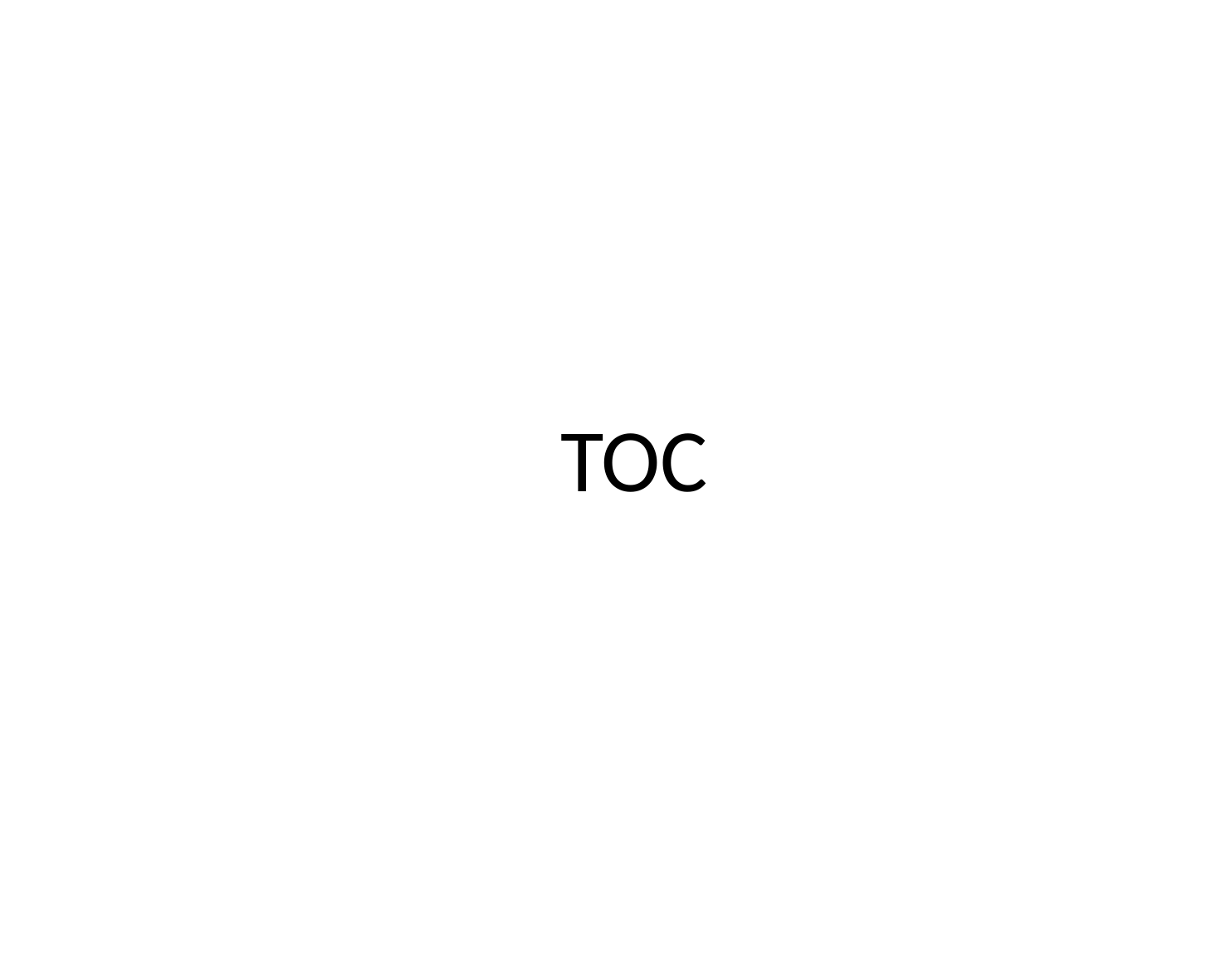

TOC

## Slide 2
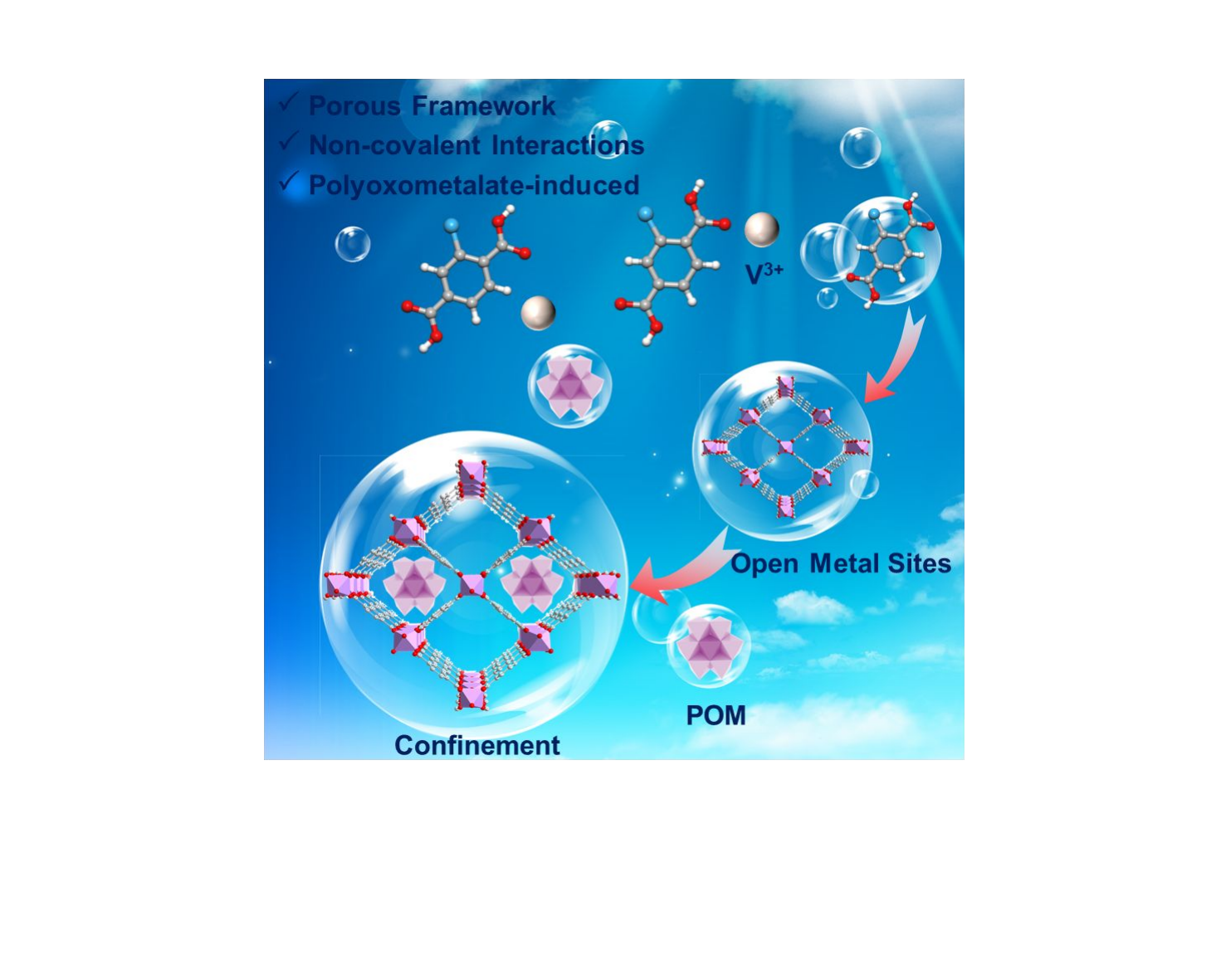

## Slide 3
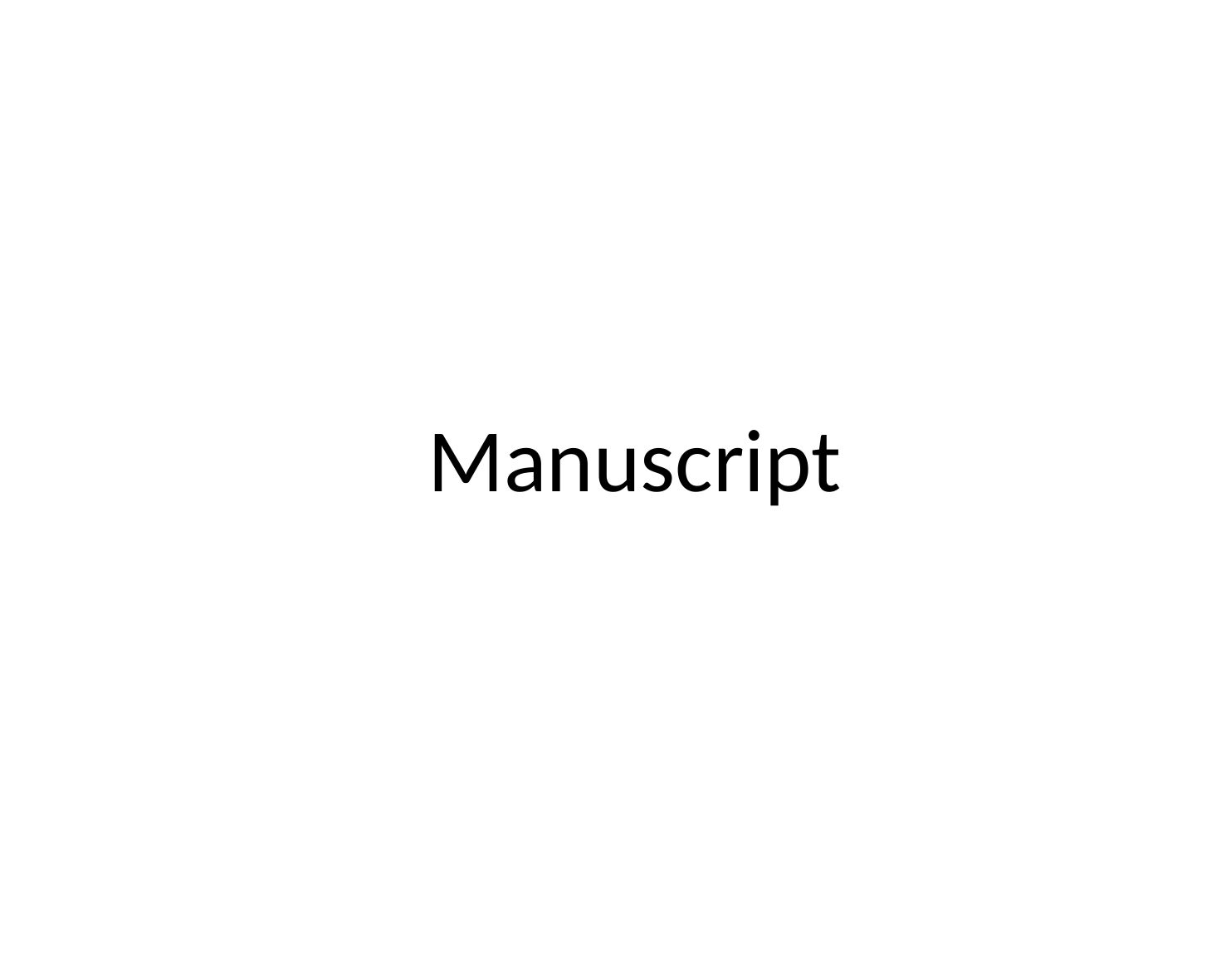

Manuscript

## Slide 4
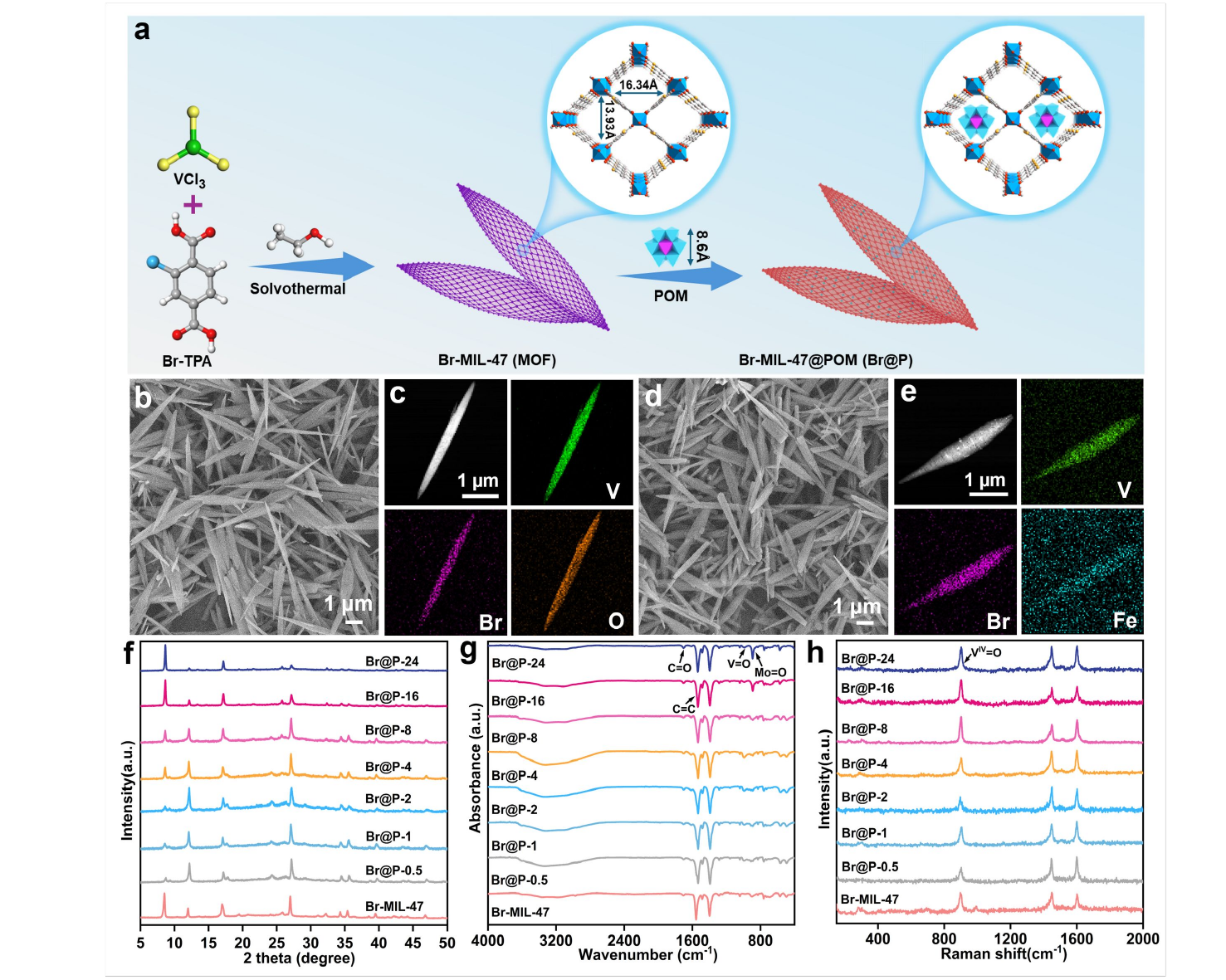

## Slide 5
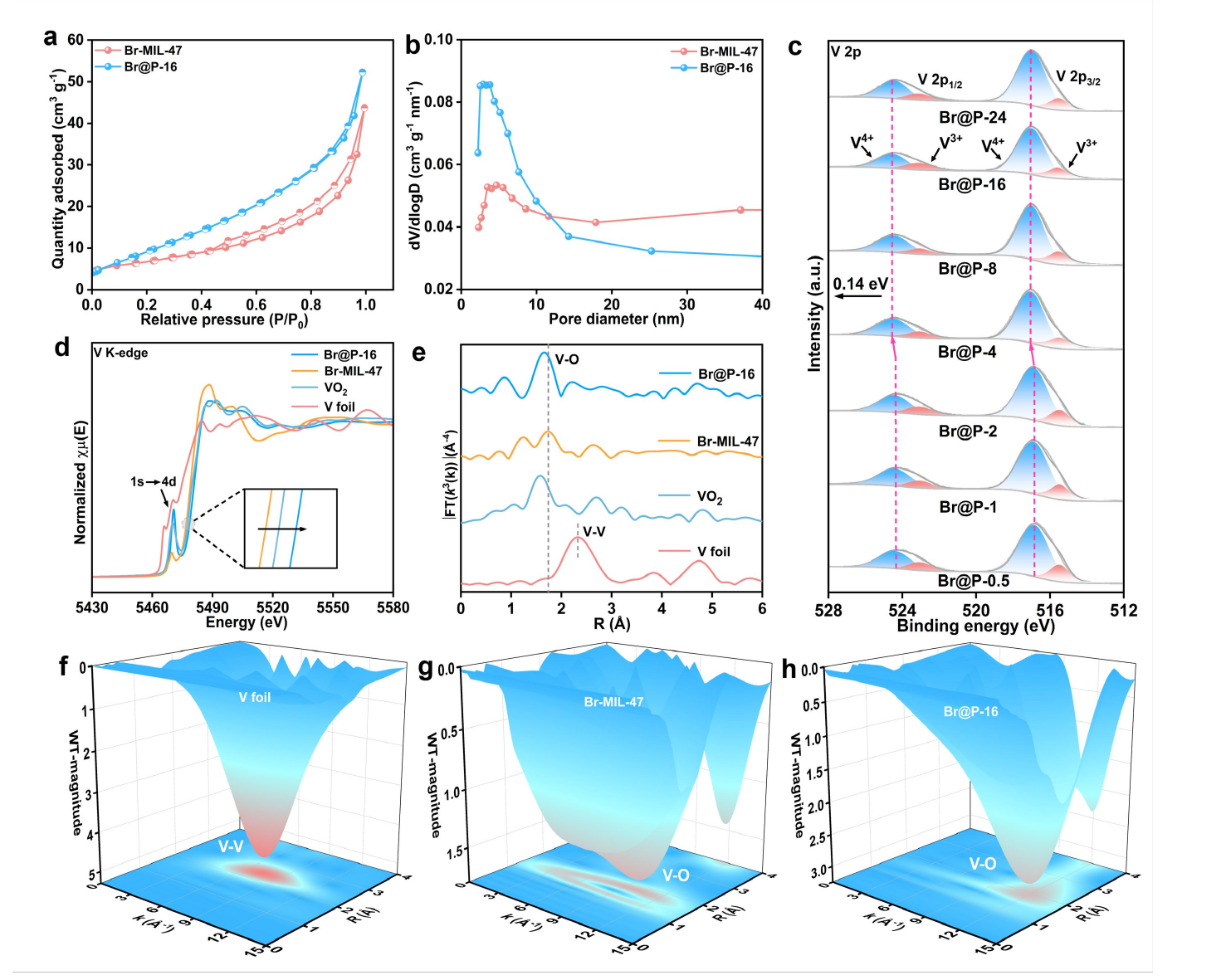

## Slide 6
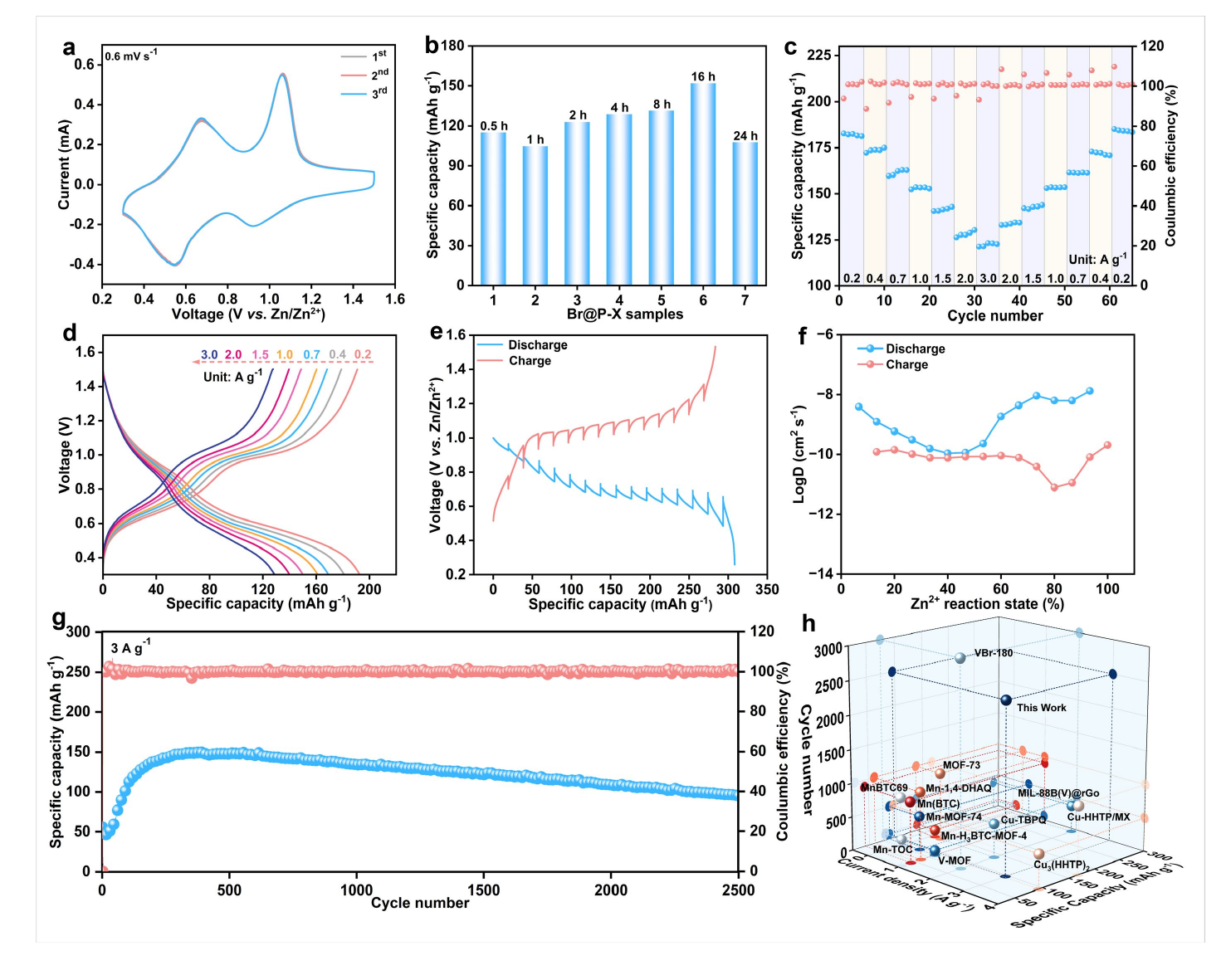

## Slide 7
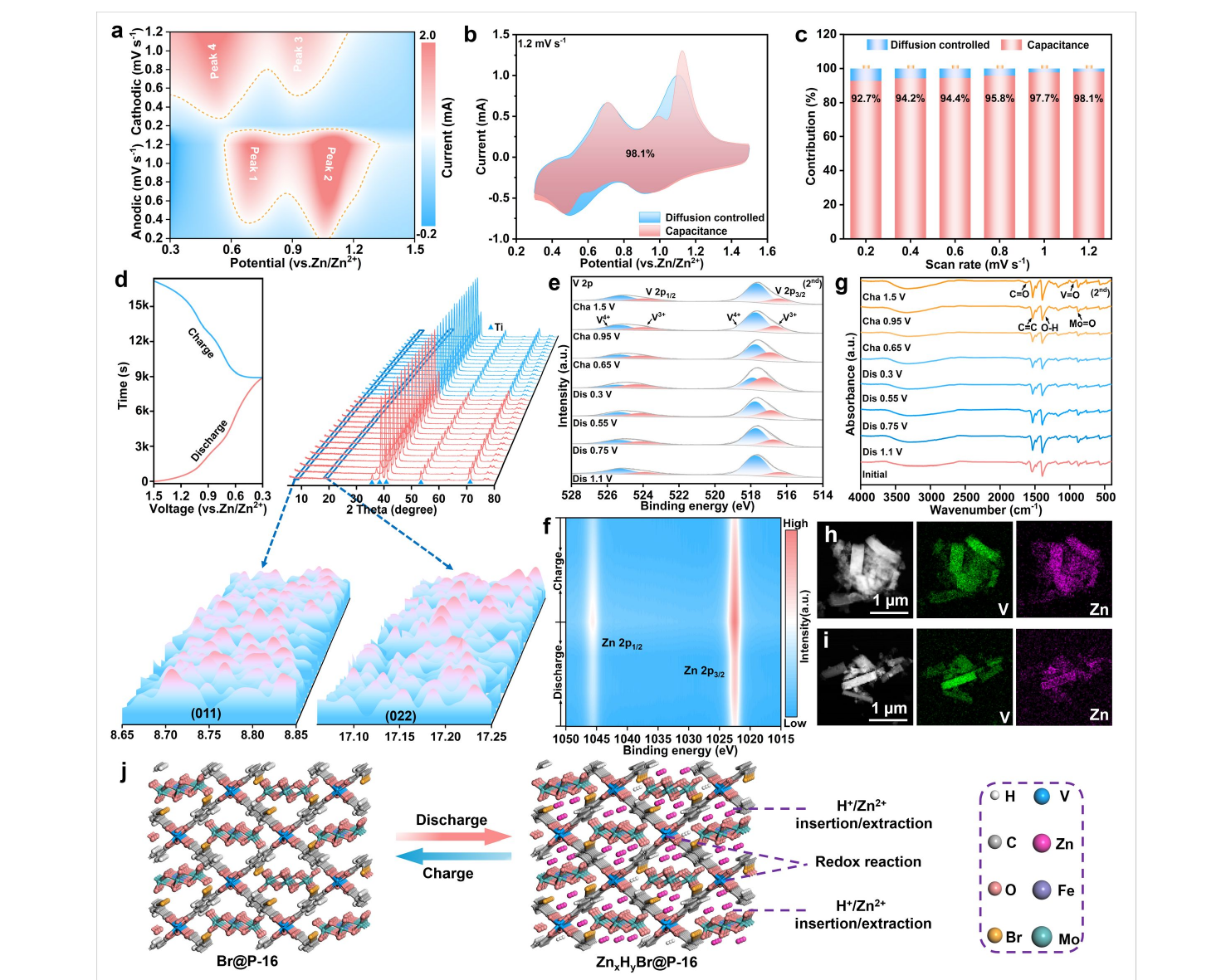

## Slide 8
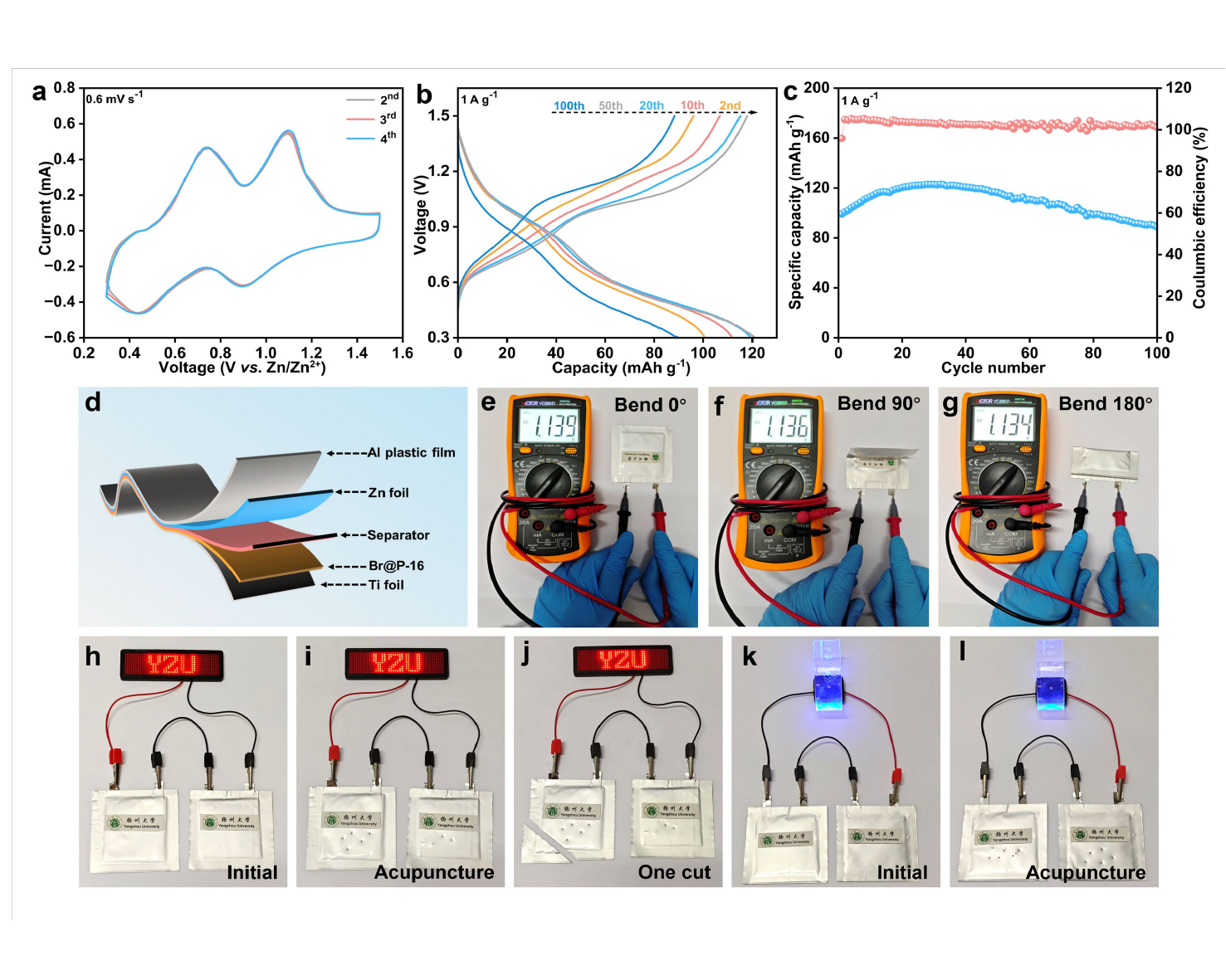

Supplement: Supplementary file 2 — Supporting Information [file ADVS-12-e11198-s002.zip › Production date/Production date.pptx]
